# Si Decoration Tuning the Electrocatalytic Activity of Ru via Turing Pattern Design

**DOI:** 10.1002/advs.202505418

**Published:** 2025-06-23

**Authors:** Chuanlong Liu, Wenfei Wang, Fangkun Sun, Xinmeng Hu, Zhilin Guo, Yijia Liu, Dongying Huang, Weizheng Cai, Guangfu Luo, Jiazhen Wu

**Affiliations:** ^1^ Department of Materials Science and Engineering Southern University of Science and Technology Shenzhen 518055 China; ^2^ Institute of Innovative Materials Southern University of Science and Technology Shenzhen 518055 China; ^3^ Guangdong Provincial Key Laboratory of Functional Oxide Materials and Devices Southern University of Science and Technology Shenzhen 518055 China

**Keywords:** electrocatalytic hydrogen evolution, intermetallic catalysts, metal surface decoration, selective chemical etching, turing pattern

## Abstract

Green hydrogen, produced via renewable‐energy‐driven water electrolysis, is among the most promising new energy carriers. Ru is a potential catalyst for this purpose; however, its strong hydrogen binding strength results in poor reaction kinetics, limiting its application potential. Creating a new Ru structure by introducing a second element is crucial for optimizing its catalytic performance. However, only a few studies have explored this approach, leaving the understanding of how chemical composition influences Ru's structure and catalytic activity elusive. Here, a systematic study is reported on Si decoration of Ru, achieving a tunable local environment around Ru and optimized reaction kinetics. By constructing a Ru‐SiO_x_ interleaved Turing‐patterned structure, the ratio of Si coordinated to Ru is tuned by well‐designed selective etching. With increasing Si content, the H‐binding strength on the Ru center is progressively weakened, resulting in a V‐shaped trend in hydrogen production activity. The optimized sample exhibits a low overpotential of 21 mV at 10 mA cm^−2^ in alkaline solution, along with a Tafel slope of 40 mV dec^−1^, surpassing the performance of commercial Pt/C. This study establishes a valuable framework for optimizing the surface properties and catalytic activity of noble metals.

## Graphical abstract

Regulating the surface properties of noble metals through the introduction of heteroatoms is crucial for designing high‐performance catalysts. By constructing a unique Ru‐SiO_x_ interleaved Turing‐patterned structure, tunable Si‐decorated Ru surfaces are achieved. Through precise, stepwise control of the Si content, the surface electronic states of Ru is optimized and enhance its catalytic performance for hydrogen evolution.

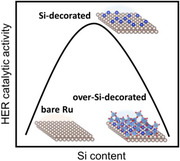

## 1 Introduction

In recent decades, with increasing energy consumption, traditional fuels such as coal, oil, and natural gas have led to increasingly urgent environmental and societal challenges.^[^ ^1^, ^2^, ^3^, ^4^ ^]^ Therefore, developing and utilizing new energy sources is crucial, among which hydrogen has garnered significant attention for its high gravimetric energy density and zero‐emission characteristics.^[^ ^5^, ^6^ ^]^ However, environmentally friendly green hydrogen, produced through water electrolysis, accounts for less than 4% of total hydrogen production and requires substantial development.^[^ ^7^, ^8^, ^9^ ^]^ One of the key challenges in water electrolysis is the cathodic hydrogen evolution reaction (HER), which requires a high externally applied potential.^[^ ^10^, ^11^, ^12^ ^]^ To address this challenge, various catalysts have been developed, including Ru/C,^[^ ^13^, ^14^ ^]^ Pt/C,^[^ ^9^, ^15^ ^]^ transition metal sulfides,^[^ ^16^, ^17^ ^]^ phosphides,^[^ ^18^, ^19^ ^]^ nitrides,^[^ ^20^, ^21^ ^]^ oxides,^[^ ^22^, ^23^ ^]^ and carbides.^[^ ^24^, ^25^ ^]^ Among these, Pt‐based catalysts are the most widely studied.^[^ ^26^ ^]^ In particular, Pt/C has been recognized as one of the most advanced HER catalysts due to its suitable hydrogen binding strength.^[^ ^9^ ^]^ However, the high cost of Pt and its relatively moderate performance in alkaline environments limit its potential for broader commercial applications.^[^ ^27^, ^28^, ^29^ ^]^

Ru, a member of the Pt‐group noble metals, holds promise as a commercially viable alternative to Pt for water electrolytic catalysts. It offers a significant cost advantage, priced at just 4% of Pt, and exhibits strong hydrogen‐binding strength (∼65 kcal mol^−1^).^[^ ^30^, ^31^ ^]^ However, this strong Ru‐H interaction limits the HER kinetics and hampers its applications.^[^ ^2^, ^32^, ^33^ ^]^ Researchers have made substantial efforts to modify Ru to enhance its catalytic performance.^[^ ^12^, ^30^, ^34^, ^35^, ^36^, ^37^, ^38^, ^39^, ^40^, ^41^, ^42^ ^]^ For instance, Ge et al.^[^ ^35^ ^]^ developed a ruthenium phosphide (RuP) film supported on carbon cloth, and X‐ray photoelectron spectroscopy (XPS) analysis confirmed electron transfer from Ru to P. This modulation by P atoms enhances the chemisorption of hydrogen on RuP, thereby improving its HER activity. Li et al.^[^ ^36^ ^]^ synthesized an amorphous ruthenium sulfide (RuS_x_) nanoparticle supported on S‐doped graphene oxide composite, functioning as a Pt‐like catalyst. The isolated Ru single‐atom sites within the amorphous RuS_x_ nanoparticles exhibit optimal Ru‐H binding energy, which is crucial to its high catalytic performance. Additionally, Cai, et al. employed Ru‐based ternary silicide electride catalysts, such as LaRuSi (LRS) and CeRuSi,^[^ ^34^, ^37^ ^]^ for HER. These modified Ru active centers demonstrated outstanding electrocatalytic activity and stability. These findings highlight the importance of regulating the electronic and local geometric structures of Ru for achieving a highly efficient Ru‐based HER catalyst. However, a comprehensive study addressing how chemical composition influences the structure and states of Ru and how these factors can be optimized for catalytic activity, remains lacking.

**Figure 1:**
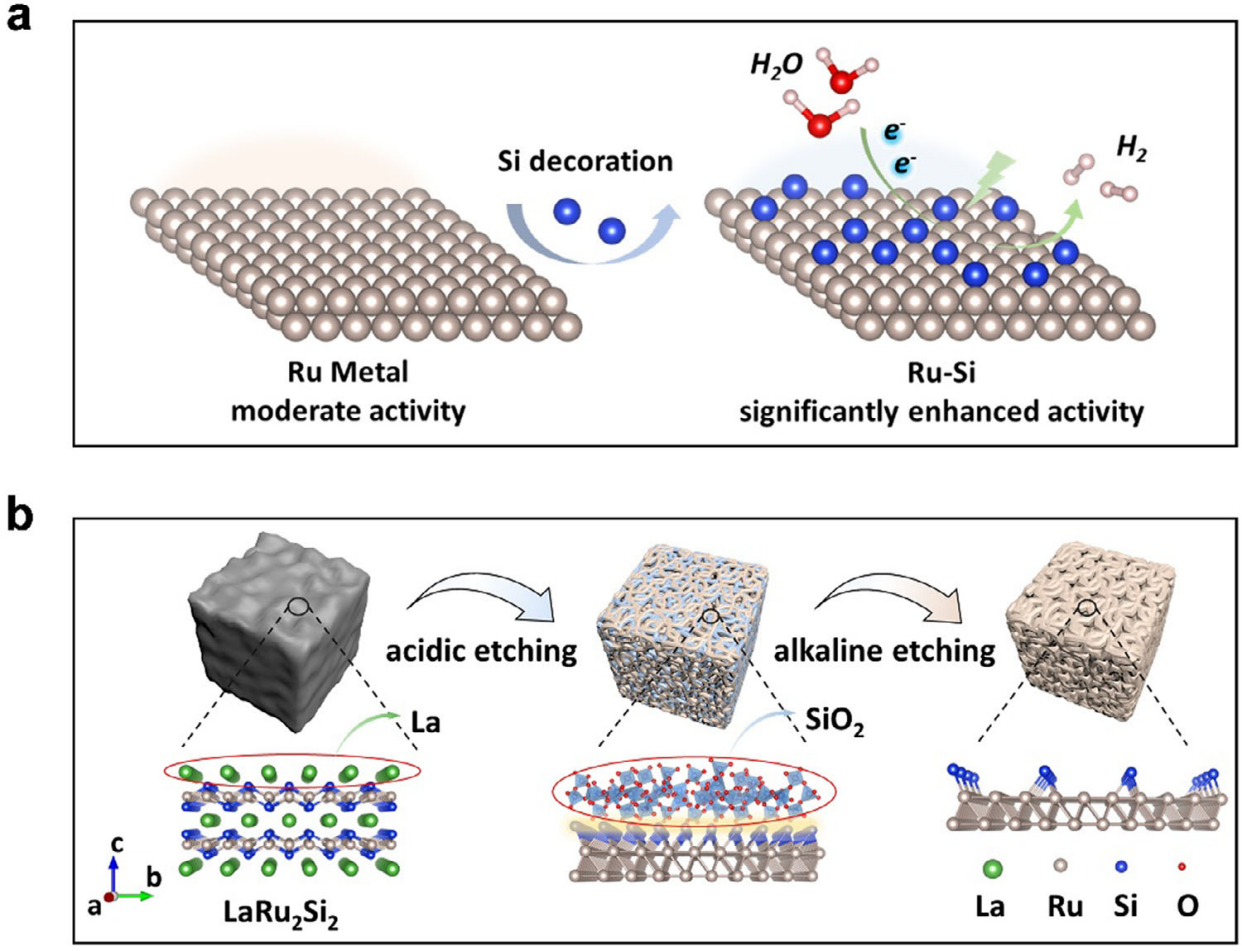
Schematic diagram of the synthetic strategy for the samples. a) Influence of Si decoration on the catalytic performance of Ru. b) Structural and compositional evolution of the Ru‐Si‐based catalysts during the selective chemical etching processes.

Herein, we present a Si‐decorated Ru system with tunable Si/Ru ratios, developed through a carefully designed selective etching method (**Figure** **1**). By precisely controlling the Si content, we optimize the alkaline electrolytic HER performance of Ru. Inspired by MXenes‐like etching processes of YRu_2_Si_2_^[^ ^43^ ^]^ and LRS,^[^ ^37^ ^]^ this work reports the discovery of Turing‐patterned Ru‐SiO_x_ structures via selective HCl etching of LaRu_2_Si_2_ (LR2S2). The resultant material features a high specific surface area (565 m^2^ g^−1^). Subsequent alkaline etching progressively removes the Si‐containing components, yielding a series of Ru catalysts with varying Si contents. These catalysts exhibit a V‐shaped activity trend as the Si content decreases. The optimal catalyst demonstrates an overpotential of only 21 mV at 10 mA cm^−2^ and a Tafel slope of 40 mV dec^−1^ in 1 M KOH. Density functional theory (DFT) calculations reveal that this V‐shaped performance trend arises from variations in H binding energy, which can be finely tuned by adjusting the Si content to optimize the *d*‐band center and charge states of Ru active sites.

## 2 Results and Discussion

### 2.1 Design Strategy for Si‐Decorated Ru Surfaces

Previous studies have shown that Ru‐Si‐containing intermetallic compounds exhibit significantly improved HER catalytic performance compared to Ru metal.^[^ ^34^, ^44^ ^]^ This suggests that Si has the potential to modify the catalytic properties of Ru. To gain a deeper understanding of how Si can alter the Ru activation center, it is essential to create surfaces with varying Si content. Therefore, we propose a Si‐decoration strategy (Figure 1a) to develop efficient and tunable Ru‐based HER catalysts. However, implementing this Si‐decoration strategy presents a challenge, as the chemical compositions and structures of reported Ru‐Si containing compounds are generally fixed and chemically stable. Additionally, incorporating Si onto Ru surface using traditional bottom‐up deposition methods is difficult. Recently, top‐down selective chemical etching methods have emerged as a promising approach to create novel Ru‐Si structures and adjust the Si/Ru rations. For example, our previous work demonstrated that La species could be selectively removed from LRS using HCl, resulting in a Ru/Si superlattice structure.^[^ ^37^ ^]^ Furthermore, Rosen et al.^[^ ^43^ ^]^ discovered a MXene‐like structure of RuSi with significantly enhanced surface area by selectively removing Y from YRu_2_Si_2_ using HF. Inspired by these studies, we turn our attention to the intermetallic compound LR2S2.

As shown in Figure 1b, LR2S2 adopts the I4/mmm space group and belongs to the tetragonal crystal system. It has a crystal structure similar to that of LRS, with La atoms and Ru‐Si atomic layers arranged in a layered fashion. Unlike LRS, which contains double La atomic layers between Ru‐Si layers (Figure S1, Supporting Information), LR2S2 features only a single La atomic layer, making it more resistant to etching compared to LRS. However, by extending the etching time to 30 days, the La species were completely removed, and a unique Ru‐SiO_x_ interleaved nanoscale Turing‐patterned structure was achieved. Turing patterns, first described by Alan Turing in 1952, arise spontaneously from differential diffusion rates and nonlinear interactions between activator and inhibitor species, forming stable spatial patterns such as stripes, spots, or hexagonal arrays.^[^ ^45^, ^46^ ^]^ In our case, the Ru‐SiO_x_ Turing pattern likely results from the differing diffusion behaviors of Ru and SiO_x_ components following La removal. Additionally, the Si species were further modified through alkaline etching, with detailed investigations provided in the following sections.

### 2.2 Material Preparation and Characterization

**Figure 2:**
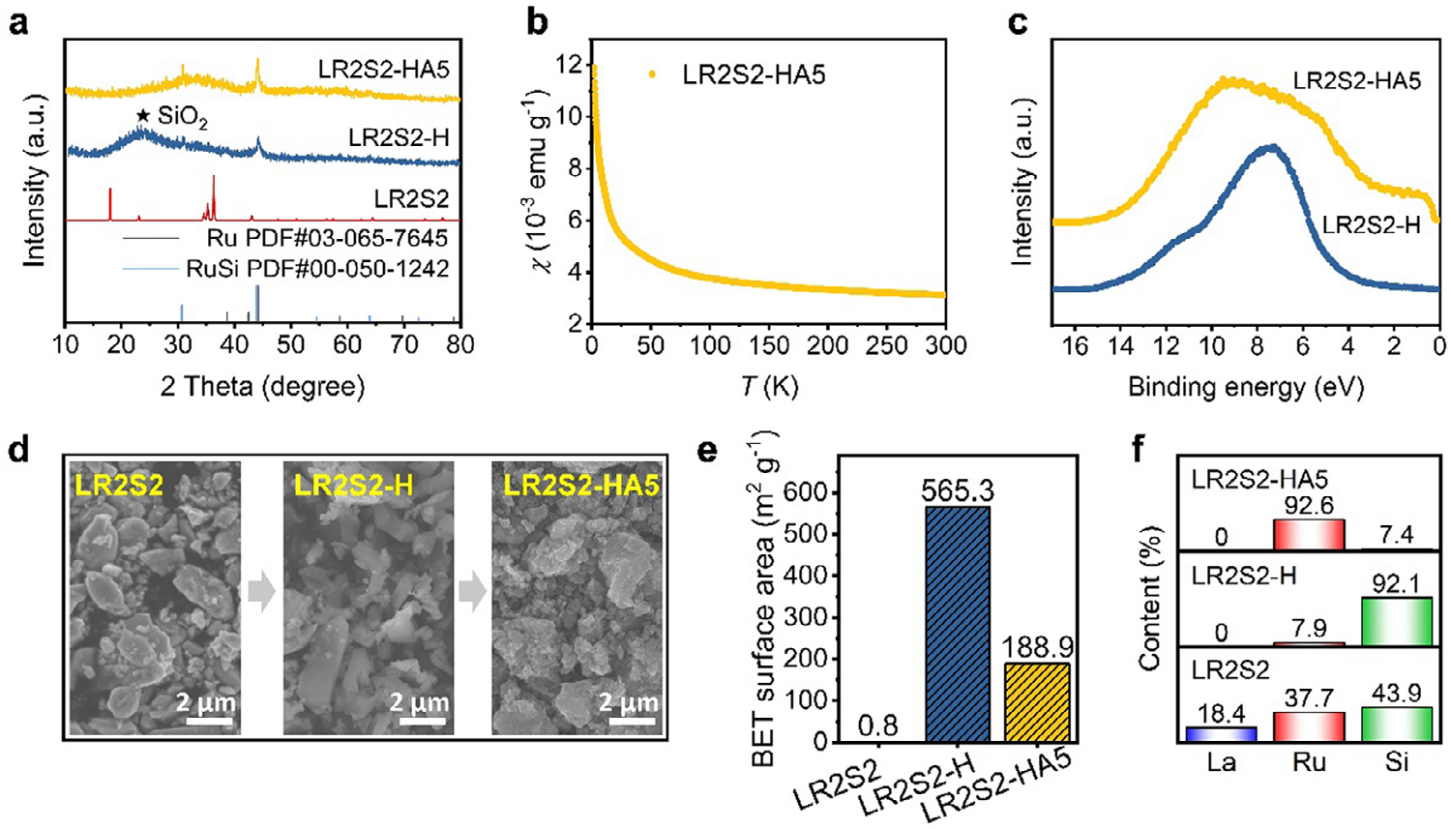
Structural, electronic, and morphological properties. a) XRD patterns of LR2S2, LR2S2‐H (acidic etching), and LR2S2‐HA5 (acidic and alkaline etching). b) Magnetic susceptibility of LR2S2‐HA5. c) UPS curves of LR2S2‐H and LR2S2‐HA5. d) SEM images of LR2S2, LR2S2‐H, and LR2S2‐HA5. e) BET surface areas. f) Elemental composition of La, Ru, and Si determined by EDS.

The phase changes before and after chemical etching were examined using X‐ray diffraction (XRD) measurements (**Figure** **2**a). After acidic etching using HCl for 30 days, the resulting LR2S2‐H sample showed a broad peak centered at 2θ  =  24°, which is primarily attributed to the amorphous SiO_2_ phase.^[^ ^47^ ^]^ The other sharp peaks are corresponding to Ru metal and RuSi intermetallic compounds. Subsequently, LR2S2‐H was subjected to further etching with 1 M KOH for 5 h, resulting in the LR2S2‐HA5 sample. After alkaline etching, the amorphous SiO_2_ peak disappeared, while the peaks for Ru metal and RuSi remained unchanged, indicating effective removal of SiO_2_. This etching process aligns with the material synthesis strategy, as schematically illustrated in Figure 1b.

Electrical conductivity is essential in electrocatalysis, as metallic materials facilitate efficient electron transfer during chemical reactions. To assess the metallic states in the prepared catalysts, magnetization measurements were conducted. As shown in Figure 2b, LR2S2‐HA5 exhibits Pauli paramagnetic behavior, likely due to conduction carriers in the sample. To verify the metallic states, ultraviolet photoelectron spectroscopy (UPS) measurements were performed. LR2S2‐HA5 shows a clear Fermi edge, confirming its metallic states (Figure 2c). In contrast, LR2S2‐H displays an insulating behavior, likely due to a significant amount of SiO_2_ coverage (Figure 2a), which can be removed through alkaline etching. This is also in accordance with the XRD results presented in Figure 2a.

The morphologies and chemical compositions of the samples were characterized using scanning electron microscopy (SEM) and energy dispersive spectroscopy (EDS). Careful analysis identified the RuSi phase, as detected in the XRD patterns, appearing as bulk structures with particle sizes exceeding 1 µm (Figure S2, Supporting Information). This particle size, comparable to that of the hand‐milled powder, indicates that RuSi was not formed during the etching process but existed as an impurity phase in LR2S2. Interestingly, SEM images (Figure 2d) reveal that the particle size and overall morphology of the samples remain largely unchanged before and after chemical etching, suggesting that the etching process primarily occurs at the microscopic level. Indeed, Brunauer–Emmett–Teller (BET) surface area measurements show a substantial increase in surface area from 0.8 m^2^ g^−1^ (LR2S2) to 565.3 m^2^ g^−1^ (LR2S2‐H) after acidic etching, attributed to the formation of a porous structure (Figure 2e; Figure S3, Supporting Information). However, the surface area decreases to 188.9 m^2^ g^−1^ (LR2S2‐HA5) following alkaline etching, likely due to the collapse of the porous microscopic structure upon removal of SiO_x_. Elemental analysis (Figure 2f; Figure S4, Supporting Information) further supports these findings. After acidic etching, the La content decreases from 18.4% to 0%, Ru decreases from 37.7% to 7.9%, and Si increases from 43.9% to 92.1%, indicating the selective removal of La and coverage of the particles with a SiO_x_ layer. Subsequent alkaline etching removes the SiO_x_, leading to a reduction in Si content from 92.1% to 7.4%, while the Ru content increases from 7.9% to 92.6%. These changes highlight the success of the etching process in modifying the material's composition, structural, and surface properties.

### 2.3 Si‐Decorated Ru Surfaces Formed within the Ru‐SiO_x_ Turing‐Patterned Structure

**Figure 3:**
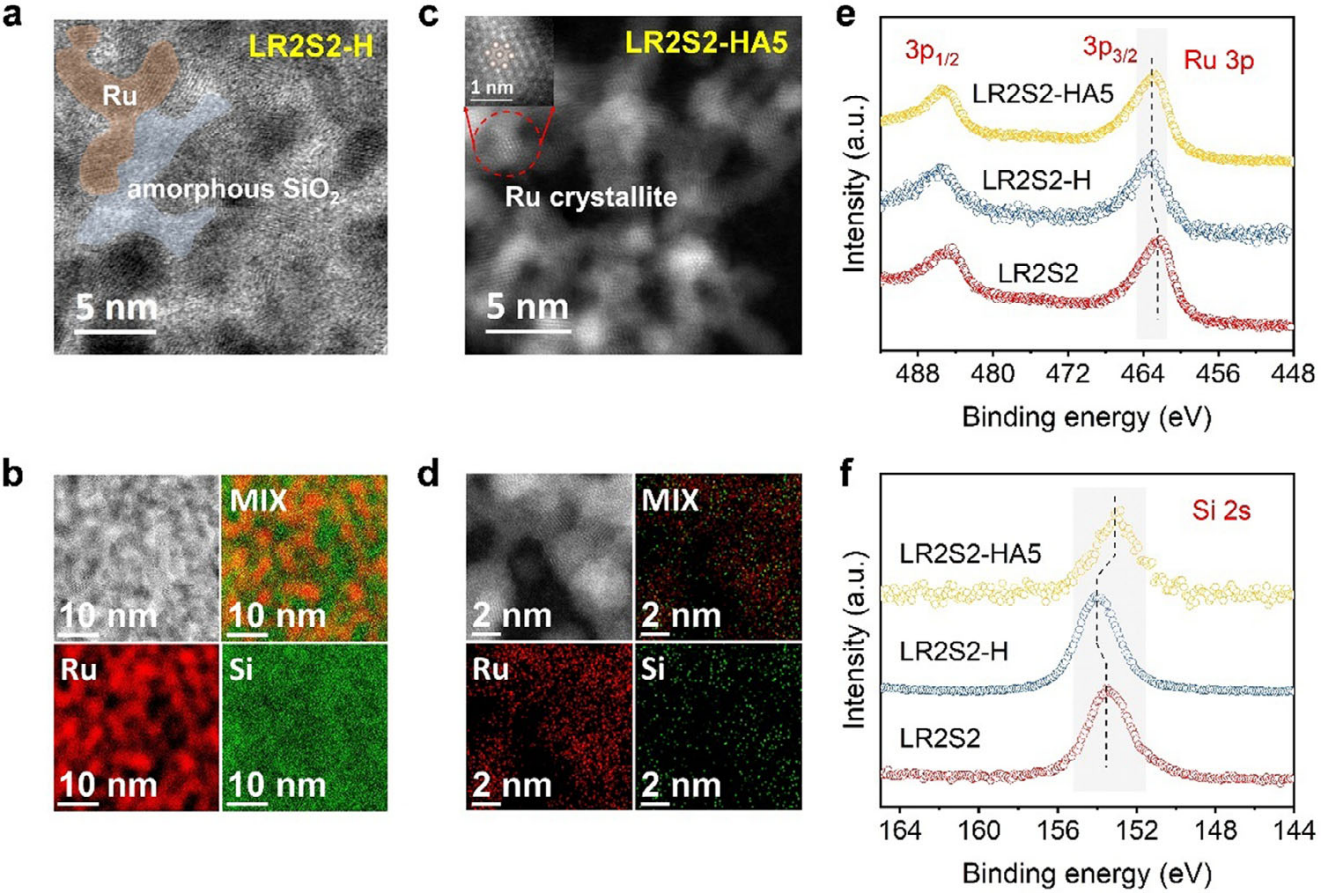
Microstructural and surface elemental characterization of Si‐decorated Ru catalysts. a) BF‐AC‐TEM image of LR2S2‐H sample. b) EDS mapping with BF‐AC‐TEM image of LR2S2‐H. c) HAADF‐AC‐TEM image of LR2S2‐HA5. d) EDS mapping with HAADF‐AC‐TEM image of LR2S2‐HA5. e) XPS spectra of Ru 3p. f) XPS spectra of Si 2s.

To investigate the microstructure of the samples, aberration‐corrected transmission electron microscopy (AC‐TEM) was performed. In the bright‐field (BF‐AC‐TEM) image of LR2S2‐H (**Figure** **3**a), two distinct regions are observed: dark areas showing Ru lattice fringes, identified as Ru regions (Figure S5a, Supporting Information), and light areas with amorphous features, primarily attributed to SiO_2_ based on XRD analysis (Figure 2a). EDS mapping (Figure 3b) further reveals an intertwined, disordered structure formed by these two phases, explaining the exceptionally high BET surface area. This structure can be categorized as a nanoscale Turing pattern, formed due to differential diffusion of Ru and SiO_x_ during selective La removal. Further studies are needed to elucidate the evolution mechanism of this pattern. Previous studies suggest such Turing patterns feature abundant interfaces, surface defects, and unsaturated ligand sites,^[^ ^48^, ^49^ ^]^ all of which enhance catalytic performance. For instance, Gu et al.^[^ ^50^ ^]^ developed Pt/Ir nanonets with a Turing structure, characterized by high specific surface area and numerous twin boundaries. DFT calculations revealed that these boundaries modulate the electronic structure of Pt, causing a downshift in the Pt *d*‐band center, which weakens hydrogen adsorption and boosts the HER performance. Similarly, the same group reported Turing‐patterned PtNiNb nanosheets,^[^ ^49^ ^]^ which also excel as HER electrocatalysts due to their high‐density twin boundaries and atomic‐level unsaturated coordination sites.

After alkaline etching, the amorphous SiO_2_ regions are nearly eliminated. The high‐angle annular dark‐field (HAADF‐AC‐TEM) image of LR2S2‐HA5 (Figure 3c) primarily shows Ru lattice fringes (Figure S5b, Supporting Information), consistent with XRD (Figure 2a) and SEM‐EDS results (Figure 2f). TEM‐EDS mapping confirms the removal of SiO_x_ (Figure 3d). However, it should be noted that Si is not entirely removed (Figure 2f) but is uniformly distributed along Ru grain boundaries, forming a unique Si‐decorated Ru surface, aligning with the proposed scheme (Figure 1).

Surface chemical characteristics of LR2S2, LR2S2‐H, and LR2S2‐HA5 were analyzed using XPS, as illustrated in Figure 3e,f. To avoid interference from overlapping Ru 3d and C 1s orbitals, the Ru 3p orbital was selected for evaluation (Figure 3e). In LR2S2, Ru exhibits a low valence state, with the Ru 3p_3/2_ peak centered at 462.5 eV, a typical value for Ru‐based intermetallic compounds.^[^ ^37^ ^]^ Following acidic and alkaline etching, the Ru 3p binding energy exhibits a slight increase, potentially attributable to mild oxidation during the etching processes. It is noteworthy that the binding energy remains unchanged between LR2S2‐H and LR2S2‐HA5, indicating that alkaline etching does not significantly alter Ru's surface electronic states. Regarding the Si 2s orbital (Figure 3f), its binding energy in LR2S2‐H is significantly higher than in LR2S2, measured at 154.1 eV, aligning with values reported for SiO_2_.^[^ ^51^, ^52^ ^]^ This confirms the observations in Figure 2, which show that SiO_2_ forms and covers the surface after acidic etching. Subsequent alkaline etching leads to a decrease in the Si 2s binding energy, suggesting that the surface SiO_2_ is largely removed and that the remaining Si exists as Ru‐Si compounds on the Ru surface. The Si 2s signal‐to‐noise ratio in LR2S2‐HA5 is significantly reduced compared to LR2S2‐H, reflecting the decreased Si content. Conversely, the Ru 3p signal‐to‐noise ratio in LR2S2‐HA5 is markedly higher than in LR2S2‐H, indicating an increased Ru content. These results are consistent with the EDS analysis (Figure 2f).

### 2.4 Catalytic Performance of the Si‐Decorated Ru Surface

**Figure 4:**
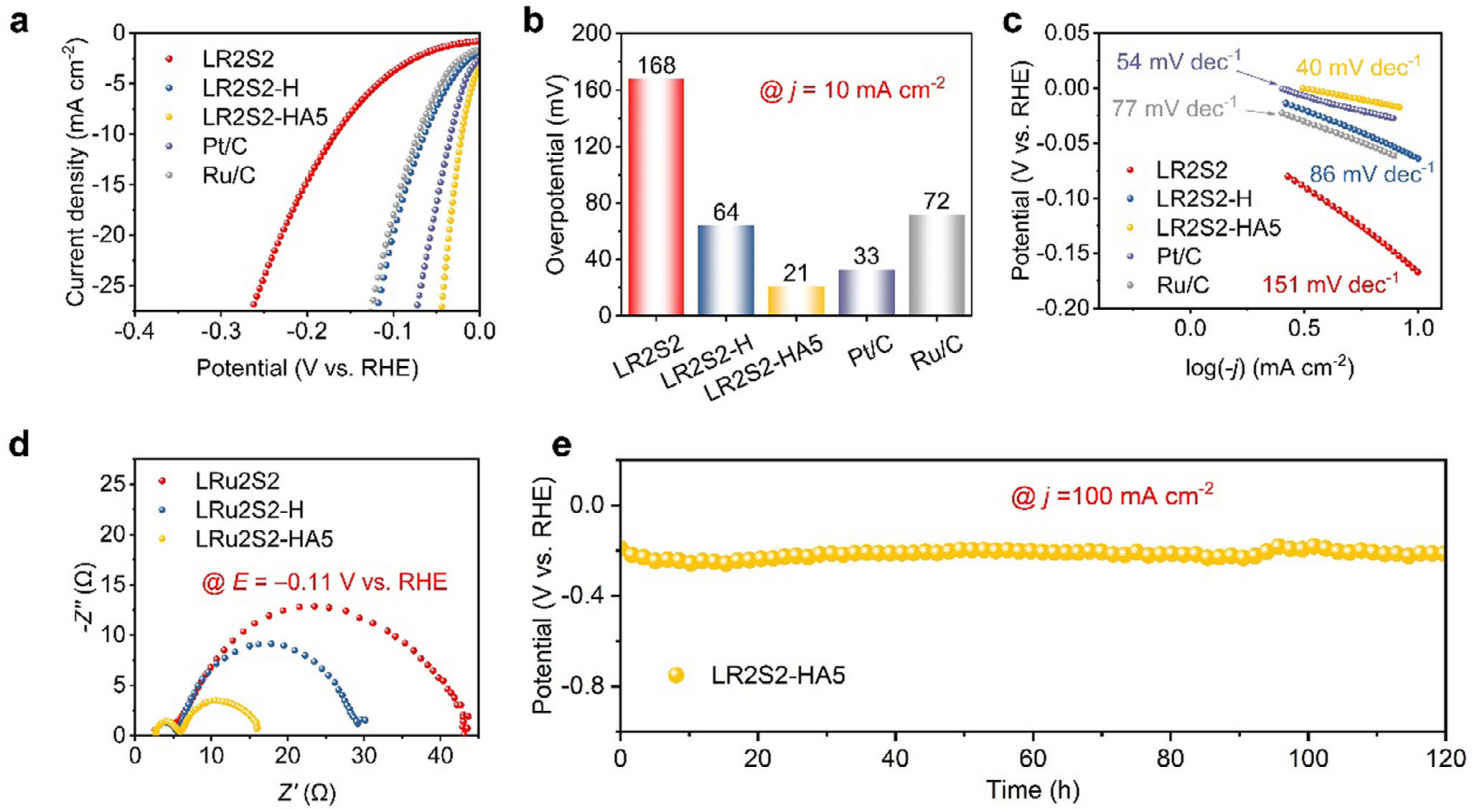
HER performance of Si‐decorated Ru catalysts. a) LSV curves for various catalysts. b) Overpotential at 10 mA cm^−2^. c) Tafel slope. d) Nyquist plots at −0.11 V versus RHE (reversible hydrogen electrode). e) Chronopotentiometry performance under a constant current density of 100 mA cm^−2^ over 120 h.

The HER experiment was conducted in a 1 m KOH solution using a standard three‐electrode setup. A glassy carbon electrode loaded with the catalyst served as the working electrode, a platinum plate (1 cm × 0.5 cm) as the counter electrode, and an Ag/AgCl electrode with a salt bridge as the reference electrode. Linear sweep voltammetry (LSV) curves were recorded to evaluate the HER activity (**Figure** **4**a). Notably, the removal of La significantly enhanced the catalytic performance of LR2S2‐H compared to LR2S2. Further removal of Si led to an additional improvement, with LR2S2‐HA5 surpassing the commercial Pt/C catalyst. At a current density of 10 mA cm^−2^, the overpotential decreased stepwise from 168 mV (LR2S2) to 64 mV (LR2S2‐H) and finally to 21 mV (LR2S2‐HA5), while the overpotential for Pt/C was 33 mV (Figure 4b).

To investigate the origin of the observed enhancement, the electrochemically active surface area (ECSA) was estimated using the double‐layer capacitance (*C_dl_*) derived from cyclic voltammetry measurements at various scan rates. Among the samples, LR2S2‐HA5 exhibited the highest *C_dl_* value of 4.91 mF, especially compared with the original LR2S2 sample (*C_dl_* = 0.17 mF), indicating a substantially enhanced ECSA (Figure S6, Supporting Information). A larger ECSA provides more active sites, which strongly benefits catalytic performance. To assess the intrinsic catalytic properties of the samples and account for current density variations due to ECSA differences, ECSA‐normalized LSV curves were calculated for LR2S2‐H and LR2S2‐HA5 (Figure S7, Supporting Information). After normalization, LR2S2‐HA5 still demonstrates markedly superior catalytic performance compared to LR2S2‐H, indicating that the enhanced performance arises primarily from improved intrinsic catalytic properties rather than solely from increased ECSA. To further investigate these intrinsic properties, Tafel slope analysis was performed (Figure 4c). With stepwise acidic and alkaline etching, the Tafel slope decreased progressively from 151 mV dec^−1^ (LR2S2) to 86 mV dec^−1^ (LR2S2‐H) and further to 40 mV dec^−1^ (LR2S2‐HA5), surpassing the 54 mV dec^−1^ of commercial Pt/C. This reduction suggests a shift in the rate‐determining step from the initial water dissociation (Volmer step) to the Volmer‐Heyrovsky mechanism.^[^ ^2^, ^18^ ^]^ To confirm the improvement in intrinsic activity, electrochemical impedance spectroscopy was conducted at −0.11 V versus RHE. As shown in Figure 4d, the Faradaic resistance, determined from the intersection of the Nyquist plot with the *Z’* axis, decreased progressively from LR2S2 to LR2S2‐H and finally LR2S2‐HA5. This trend indicates enhanced electron transfer efficiency, which is beneficial for electrocatalytic reactions.

As discussed earlier, a small amount of RuSi impurity is present in the LR2S2 sample and persists throughout the etching process (Figure 2a; Figure S2, Supporting Information). To rule out RuSi as the active catalytic component, a pure RuSi sample was synthesized and evaluated for HER performance. The synthesized RuSi particles (Figure S8, Supporting Information) display morphologies comparable to those of the RuSi impurity phase (Figure S2, Supporting Information). The catalytic performance, shown in Figure S9 (Supporting Information), reveals that the overpotential and Tafel slope of RuSi are significantly larger than those of the etched LR2S2 series samples. This indicates that the performance enhancements observed after etching are unrelated to the presence of the RuSi impurity.

Stability is as crucial as catalytic activity for a catalyst, as it determines its potential for practical applications. To assess stability, LR2S2‐HA5 was loaded onto carbon paper and subjected to a 120‐h durability test at a current density of 100 mA cm^−2^. During the test, the catalyst exhibited excellent stability with no significant increase in overpotential (Figure 4e). Furthermore, the XRD patterns before and after the durability test showed no discernible changes (Figure S10, Supporting Information), confirming the remarkable chemical stability of the sample.

### 2.5 Tuning the Catalytic Activity of Ru by Varying Si Content

The above analysis indicates that the etching process improves both the quantity and intrinsic activity of active sites. To investigate further, samples with varying Si ratios were systematically prepared by controlling the alkaline etching time. Specifically, LR2S2‐HA1, LR2S2‐HA3, LR2S2‐HA5, and LR2S2‐HA24 were obtained by etching in 1 m KOH for 1, 3, 5, and 24 h, respectively. To achieve a higher degree of etching, the alkaline concentration was increased to 6 m and the etching duration was extended to 3 days, yielding the LR2S2‐HAx sample. EDS analysis determined the specific Si ratios as 27.2% (LR2S2‐HA1), 12.4% (LR2S2‐HA3), 7.4% (LR2S2‐HA5), 2.3% (LR2S2‐HA24), and 1.5% (LR2S2‐HAx). Notably, XRD patterns showed no significant structural changes across the samples (Figure S11, Supporting Information), confirming that alkaline etching selectively reduced Si content without affecting the overall structural framework.

**Figure 5:**
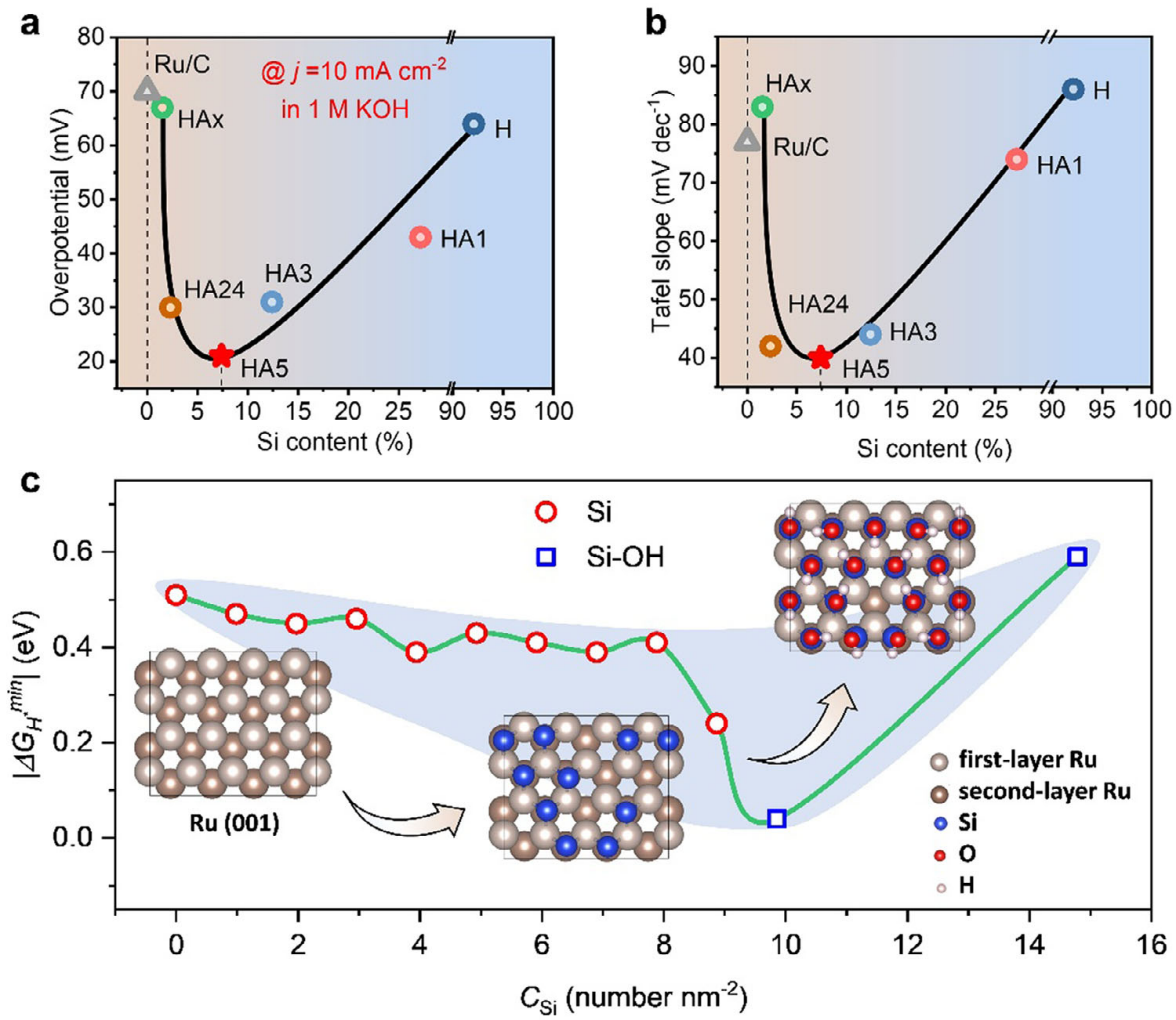
Catalytic mechanism analysis of Si‐decorated Ru catalysts. a) Relationship between overpotential and Si content at 10 mA cm^−2^. b) Relationship between Tafel slope and Si content. c) Absolute value of H adsorption energy as a function of Si concentration, determined by DFT calculations. The shaded area serves as a visual guide.

The relationship between overpotential at a current density of 10 mA cm^−2^ and Si content (as determined by EDS), as well as between Tafel slope and Si content, are depicted in **Figure** **5**a (Figure S12a, Supporting Information) and Figure 5b (Figure S12b, Supporting Information), respectively. Interestingly, as the Si content decreases due to progressive etching, the overpotential initially declines and then rises, with the Tafel slope exhibiting a similar trend. This behavior highlights the influence of Si decoration on enhancing the catalytic performance of Ru. Optimal catalytic activity is achieved when the Si content is approximately 7.4% (LR2S2‐HA5). Its catalytic performance surpasses that of most recently reported Ru‐based catalysts (Table S1, Supporting Information). Moreover, it is worth noting that the performance of LR2S2‐HAx, in both overpotential and Tafel slope, resembles that of Ru/C. This similarity can be attributed to the minimal Si content (1.5%) in LR2S2‐HAx. Accounting for the presence of the RuSi impurity, the effective Si content on the Ru surface is nearly zero, leading to LR2S2‐HAx exhibiting characteristics comparable to those of pure Ru.

To explain the experimentally observed V‐shaped HER performance trend with varying Si concentrations, DFT calculations were carried out. The Ru (001) surface was selected for study due to its lowest surface energy. Si‐decorated Ru surfaces were modeled by placing Si atoms at the most stable adsorption sites on the Ru (001) surface (Figures S13,S14, Supporting Information). Based on the valence states of Si identified through XPS analysis (Figure 3f), two models were used: Si atoms for low Si concentrations and SiOH functional groups for higher concentrations. Hydrogen adsorption energies were calculated for various Ru sites on these Si‐decorated surfaces (Figure S15, Supporting Information). The results reveal that bare Ru (001) surface exhibits the strongest hydrogen adsorption, which weakens gradually as Si concentration increases. At nearly complete surface coverage with SiOH, the hydrogen adsorption becomes highly unfavorable, yielding a large positive binding energy. The absolute values of the lowest hydrogen binding energies for different Si‐decorated Ru surfaces are summarized in Figure 5c, forming a V‐shaped trend. This indicates that moderate Si decoration optimizes hydrogen adsorption strength (approaching zero), aligning with the V‐shaped catalytic performance observed in Figure 5a,b. These findings suggest that Si decoration is an effective strategy for enhancing Ru‐based HER catalysts, with the potential to replace Pt in practical applications.

To further investigate the optimization mechanism, the *d*‐band center and surface charge states of Ru active sites were analyzed. As shown in Figure S16 (Supporting Information), increasing Si coverage leads to a monotonic decrease in the *d*‐band center from −2.05 eV for pristine Ru to −2.88 eV at a Si coverage of 14.79 Si nm^−2^. A lower *d*‐band center is known to weaken interactions between *d* electrons and adsorbates, suggesting that higher Si coverage reduces hydrogen adsorption strength. This trend is also corroborated by Bader charge analysis (Figure S17, Supporting Information), which reveals that surface Ru atoms become increasingly negatively charged with higher Si coverage, indicating a diminished capacity to accept electrons from adsorbed hydrogen and thus weaker hydrogen adsorption. These results consistently explain the observed trend in Figure S15 (Supporting Information), where hydrogen adsorption energy increases with Si content. Thus, the optimized hydrogen adsorption strength primarily results from tailored electronic states of Ru active sites induced by Si decoration. It is noteworthy that surface defects and unsaturated coordination sites may also contribute to the enhanced catalytic performance, as noted in prior studies.^[^ ^53^, ^54^, ^55^, ^56^ ^]^ However, the amorphous surface structure of the current catalyst complicates quantitative analysis of these factors. Further research is needed to address this challenge.

## 3 Conclusion

In summary, we developed a selective etching strategy that revealed a distinctive Turing‐patterned Ru‐SiO_x_ structure and Si‐decorated Ru surfaces derived from the intermetallic compound LR2S2. The resulting material exhibits tunable Si concentrations, high specific surface area, and excellent metallic conductivity. HER catalytic activity displayed a V‐shaped trend with decreasing Si content, with the optimized catalyst achieving a Si concentration of ≈7.4% (LR2S2‐HA5). This catalyst demonstrated exceptional performance, achieving an overpotential of only 21 mV at 10 mA cm^−2^ and a Tafel slope of 40 mV dec^−1^ in 1 M KOH, outperforming commercial Pt/C catalysts. DFT calculations attributed the V‐shaped performance to variations in hydrogen binding energy induced by different Si decorations on Ru active sites. Optimal hydrogen adsorption strength is achieved by tuning the *d*‐band center and charge states of Ru through varying Si coverage. The present study not only introduces an efficient Ru‐based HER catalyst but also provides a valuable framework for tailoring surface properties and catalytic activity for noble metals.

## Conflict of Interest

The authors declare no conflict of interest.

## Author Contributions

J.W. proposed the idea and supervised the research. C.L., W.C., F.S., X.H., Z.G., Y.L., and D.H. conducted the materials synthesis and characterization. W.C. and C.L. performed the electrochemical experiments, and analyzed the experimental data. W.W. and G.L. performed the DFT calculations. C.L., W.C., G. L., and J.W. co‐wrote the paper. All authors discussed the results and commented on the manuscript.

## Supporting information



Supporting Information

## Data Availability

The data that support the findings of this study are available from the corresponding author upon reasonable request.;
