# Supplementary material for: Si Decoration Tuning the Electrocatalytic Activity of Ru via Turing Pattern Design
Source: Adv Sci (Weinh). 2025 Jun 23;12(35):e05418. doi: 10.1002/advs.202505418 (PMC12462982; doi:10.1002/advs.202505418)
Supplement: Supplementary file 1 — Supporting Information [file ADVS-12-e05418-s001.docx]

**Supporting Information**

**Si Decoration Tuning the Electrocatalytic Activity of Ru via Turing Pattern Design**

*Chuanlong Liu^1,4^, Wenfei Wang^1,4^, Fangkun Sun^1^, Xinmeng Hu^1^, Zhilin Guo^1^, Yijia Liu^1^, Dongying Huang^1^, Weizheng Cai^1,*^, Guangfu Luo^1,2,*^, Jiazhen Wu^1,2,3,*^*

^1^ Department of Materials Science and Engineering, Southern University of Science and Technology, Shenzhen, China.

^2^ Institute of Innovative Materials, Southern University of Science and Technology, Shenzhen, China.

^3^ Guangdong Provincial Key Laboratory of Functional Oxide Materials and Devices, Southern University of Science and Technology, Shenzhen, China.

^4^ These authors contributed equally: Chuanlong Liu, Wenfei Wang.

^*^ E-mail: caiw0014@e.ntu.edu.sg; luogf@sustech.edu.cn; wujz@sustech.edu.cn

**Experimental Section**

*Chemicals:* All chemicals were purchased and used without further purification. La (99.9%) and Si (99.999%) were purchased from Thermo Scientific, Ru (99.95%) was purchased from PrMat Co., Ltd. Potassium hydroxide (semiconductor grade, 99.99% trace metals basis) and Nafion 117 solution (around 5% in a mixture of lower aliphatic alcohols and water) were purchased from Sigma-Aldrich. Ruthenium on carbon (Ru/C, Ru 5%) was purchased from Macklin Biochemical Co. Ltd. Platinum on activated carbon (Pt/C, Pt 20%) was purchased from Innochem.

*Synthesis of LR2S2 and RuSi:* LR2S2 and RuSi were synthesized via an arc-melting method employing stoichiometric amounts of lanthanum, ruthenium, and silicon ingots under an argon atmosphere. The melting process was repeated six times to ensure homogeneity, with a weight loss of less than 1%. The obtained ingots showed a silver color and were manually pulverized in an agate mortar under ambient air conditions.

*Synthesis of LR2S2-H:* LR2S2-H was prepared by selectively removing La atoms through chemical etching with concentrated hydrochloric acid. Specifically, 3.5 g LR2S2 powder was placed in a clean beaker, followed by the addition of 80 mL of 6 M HCl. The mixture was stirred at 600 rpm at room temperature for 30 days and then centrifuged to separate the resulting LR2S2-H. The product was washed six times with distilled water and dried under vacuum at room temperature for 12 hours.

*Synthesis of LR2S2-HAn (n = 1, 3, 5, 24, x):* LR2S2-HAn (n = 1, 3, 5, 24, x) was synthesized by further removing surface SiO_x_ through aqueous alkaline etching. For LR2S2-HA1 to LR2S2-HA24, 1 M KOH was used as the etchant. Specifically, 10 mL of the etchant was placed into a clean Teflon-lined hydrothermal reactor containing 0.1 g of LR2S2-H powder, and the reactor was heated in an electric blast drying oven at 70 °C for 1, 3, 5, or 24 hours, respectively. For LR2S2-HAx, a more concentrated alkaline solution (6 M NaOH) was used as the etchant to completely remove the Si component from the sample. Specifically, 30 mL of the etchant was added to a clean beaker containing 0.45 g of LR2S2-H powder, and the mixture was stirred at 600 rpm at room temperature for 3 days under ambient air. After etching, the LR2S2-HAn (n = 1, 3, 5, 24, x) catalysts were then separated by centrifugation, washed six times with distilled water, and dried in a vacuum oven at room temperature for 12 hours.

*Materials Characterization:* XRD measurements were performed using an X-ray powder diffractometer with Cu K$\alpha$ radiation (Bruker D2 PHASER). The morphology of the sample was examined using field emission scanning electron microscopy (FESEM, HITACHI SU8230), and the elemental composition was analyzed using EDS. BET specific surface area of the samples was determined from nitrogen adsorption-desorption isotherms at −196 °C using an automatic gas adsorption instrument (Micromeritics, ASAP2460). Magnetic properties were measured using a magnetic properties measurement system (MPMS-3, Quantum Design Inc.). AC-TEM images were acquired with a Thermo Themis Z transmission electron microscope operating at 200 kV. The surface chemical information was obtained through XPS using an ESCALAB Xi+ photoelectron spectrometer (Thermo Scientific) with a monochromatic Al K$\alpha$ X-ray beam (1486.6 eV). All binding energies were referenced to the C 1s peak (284.8 eV).

*Electrochemical Measurements:* Electrochemical measurements were performed using a CHI 760E electrochemical workstation at room temperature with a typical three-electrode system. A rotating disk glassy carbon electrode (PINE) with a diameter of 5 mm and an area of 0.196 cm^2^ was used as the working electrode. Platinum wire and Ag/AgCl electrodes (PINE, saturated with KCl and calibrated against a standard hydrogen electrode) served as the counter and reference electrodes, respectively. To minimize the influence of the electrolyte, a salt bridge was employed between the alkaline media and the Ag/AgCl electrode during all electrocatalytic experiments. The potential was calculated using Eq. (1):

$E(RHE) = E(Ag/AgCl) + 0.197+ 0.0592 \times\mathrm{pH}$ (1)

The working electrode on glassy carbon was prepared as follows. First, 5 mg of the catalyst powder was dispersed in a mixture of deionized water (480 $\mu$L), isopropanol (480 $\mu$L) and Nafion 117 solution (40 $\mu$L) and ultrasonicated for 1 hour. The resulting catalyst ink suspension (10 $\mu$L) was then pipetted onto a pre-cleaned glassy carbon disk electrode, achieving a catalyst loading of 250 $\mu$g/cm^2^. Finally, the glassy carbon electrode was air-dried at room temperature for over 1 hour.

Prior to electrochemical data collection, the working electrodes underwent continuous potential cycling between $-$0.04 and 0.2 V vs. RHE until reproducible voltammograms were obtained (room temperature, 1600 rpm, and a scan rate of 100 mV/s). The solution resistance (*R_s_*) was determined from the resulting Nyquist plot and was used to correct for the Ohmic drop, using the equation *E_c_* = *E_m_* − *iR_s_*, where *E_c_* is the corrected potential and *E_m_* is the measured potential. To evaluate the HER activity, linear sweep voltammetry (LSV) was conducted at a scan rate of 10 mV/s in 1 M KOH.

Electrochemical impedance spectroscopy (EIS) measurements were conducted using the same CHI 760E electrochemical workstation. The EIS spectra were recorded by applying various DC potentials with a frequency range from 0.1 Hz to 100 kHz. A sinusoidal amplitude of 10 mV was applied to ensure a linear response of the electrode.

The electrochemically active surface area (ECSA) was estimated using the double-layer capacitance method. First, the double-layer charging potential region was determined from a static CV scan. The charging current *i_c_* was calculated from the CV curves obtained at different scan rates. The relationship between *i_c_*, scan rate (*v*) and *C_dl_* is given by *i_c_* = *vC_dl_*. The ECSA can then be estimated using the equation ECSA = *C_dl_*/*C_s_*, where *C_s_* is the ideal specific capacitance of a smooth planar surface, set as a constant in this work.

*Computational Details:* All first-principles calculations were performed based on density functional theory (DFT) as implemented in the Vienna *Ab initio* Simulation Package (VASP)^[1]^. The Perdew-Burke-Ernzerhof (PBE)^[2]^ exchange-correlation functional was employed, along with the D3 dispersion correction scheme to account for van der Waals interactions^[3]^. Plane-wave cutoff energies of 294 eV and 339 eV were used for systems without and with oxygen, respectively. Projector-augmented-wave (PAW) pseudopotentials^[4]^ were used, with Ru(4d^7^5s^1^) for Ru, Si_GW(3s^2^3p^2^) for Si, O_s(2s^2^2p^4^) for O, and H ultrasoft (1s^1^) for H. Structure optimizations were carried out until the total energy change and the remaining force per atom were less than 10^−5^ eV and 0.04 eV/Å, respectively. A Γ-centered k-point mesh with a spacing of 2π/40 Å^−1^ was used for the two in-plane directions in the Brillouin zone. The supercells consisted of four Ru layers, with the bottom two fixed in their positions to simulate the bulk, and the in-plane dimensions were 10.82 $\times$ 9.37 Å^2^. A vacuum slab of 10 Å was added in the out-of-plane direction to reduce image interactions. The hydrogen binding energy (*G_b_*) on each substrate is defined by Eq. (2):

$G_{b}=G\left( *H \right)-G\left( * \right)- 1/2G(H_{2})$, (2)

where *G* ($*$), *G*($*$H), and *G*(H_2_) represent the Gibbs free energies of the substrate, the substrate with adsorbed hydrogen, and H_2_ gas, respectively. The temperature was set to 298.15 K and the partial pressure of H_2_ was 1 atm.


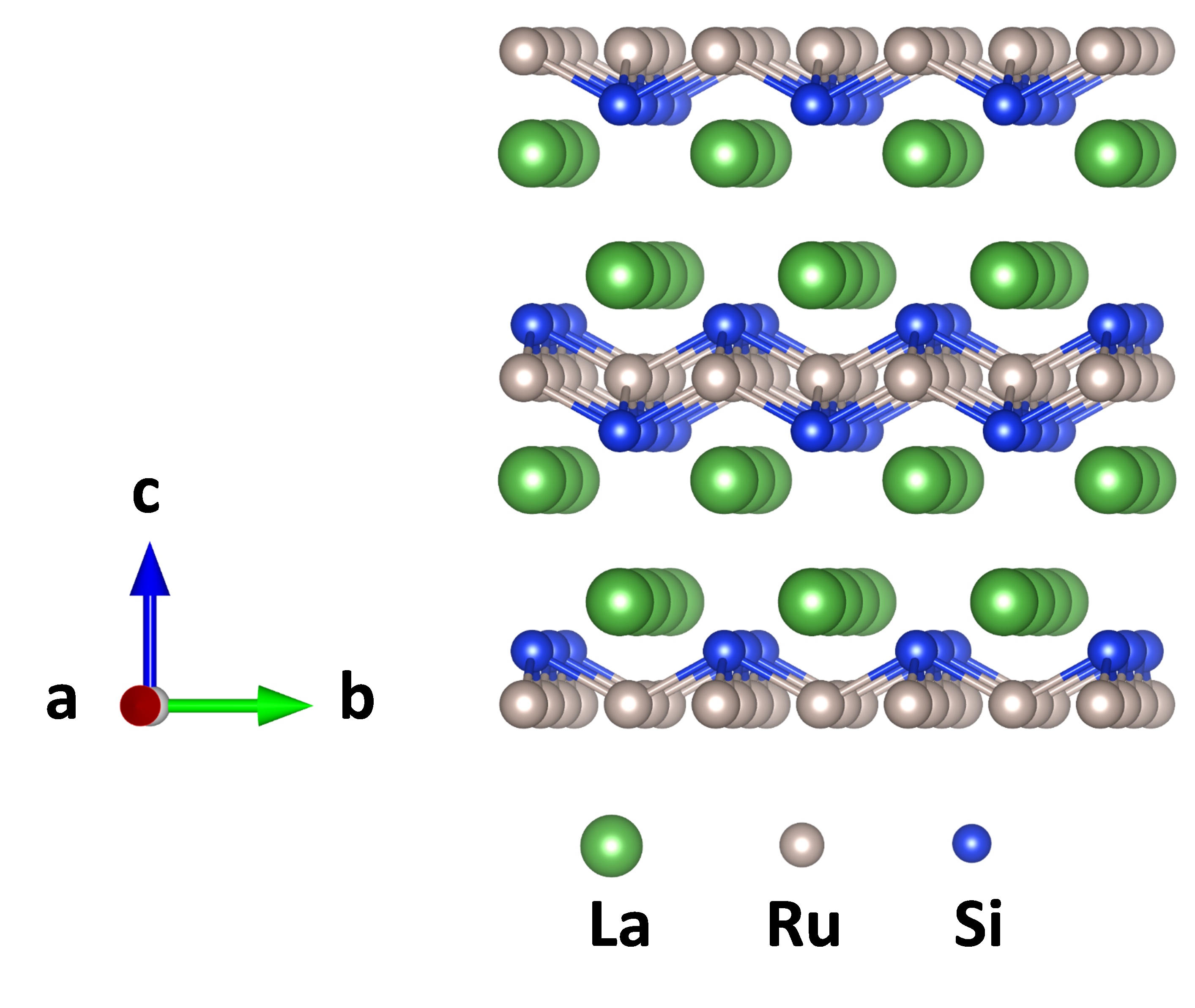


**Figure S1.** Crystal structure of LaRuSi.


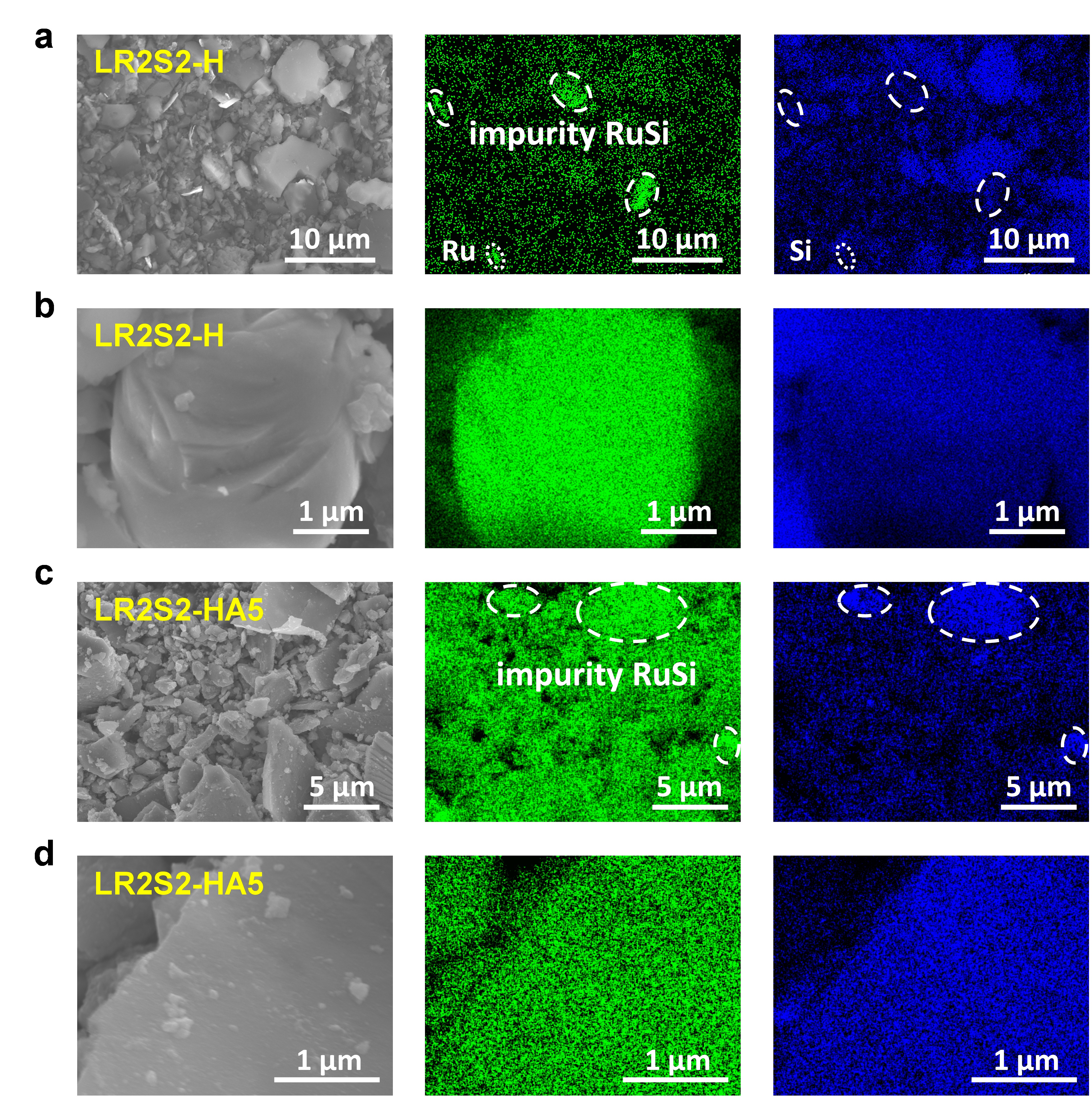


**Figure S2.** Identification of RuSi impurities by SEM-EDS measurements. a) & b) LR2S2-H, c) & d) LR2S2-HA5.


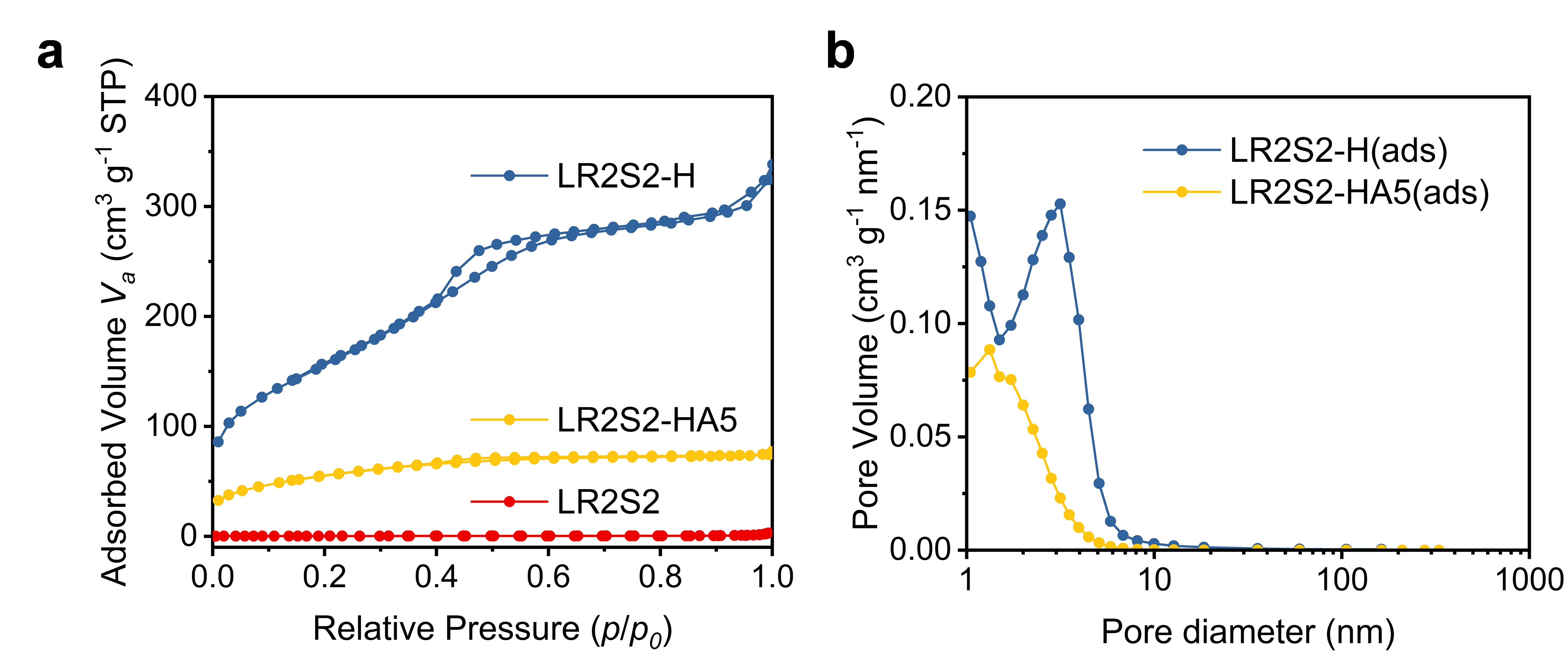


**Figure S3.** The Brunauer–Emmett–Teller (BET) analysis. a) N_2_ adsorption-desorption isotherms. b) Barret-Joyner-Halenda (BJH) pore size distribution curves.


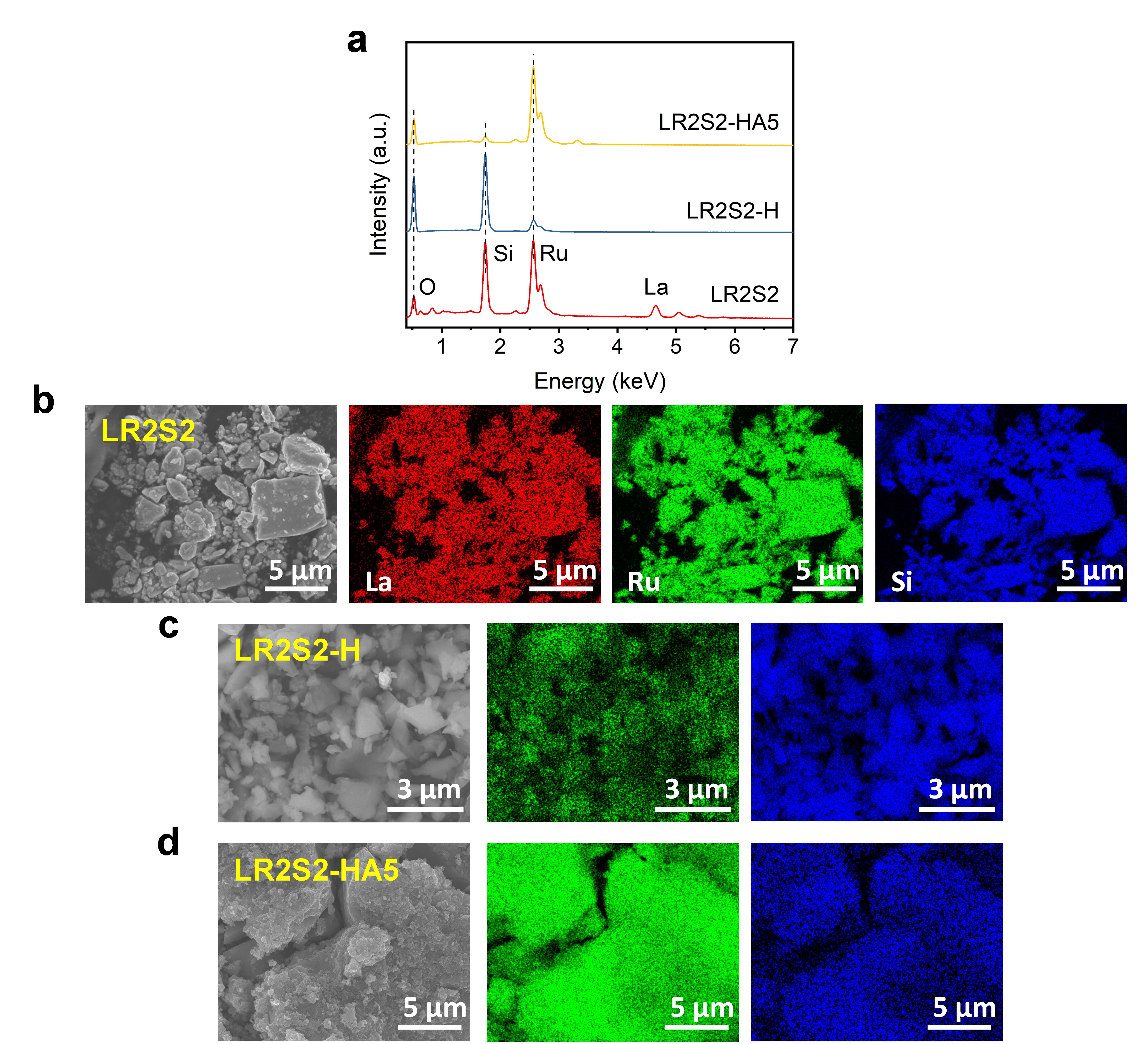


**Figure S4.** Energy dispersive X-ray spectra (EDS) elemental analysis. a) EDS spectra. b) EDS Mapping of LR2S2. c) EDS Mapping of LR2S2-H. d) EDS Mapping of LR2S2-HA5.


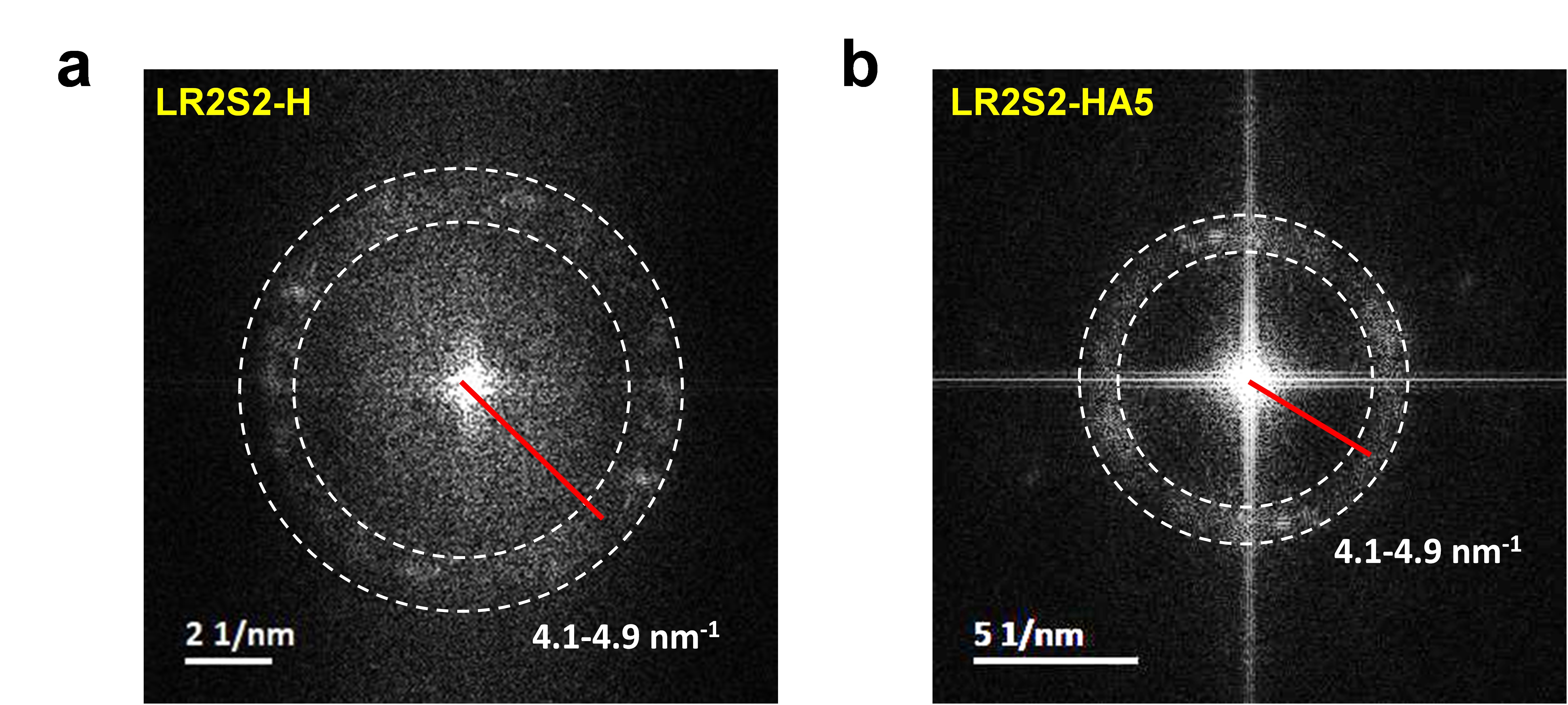


**Figure S5.** Fast Fourier transform (FFT) patterns of AC-TEM images. a) LR2S2-H. b) LR2S2-HA5.

Note: The ring width ranges from 4.1 to 4.9 nm^−1^, corresponding to the (100), (002), and (101) crystal planes of Ru.


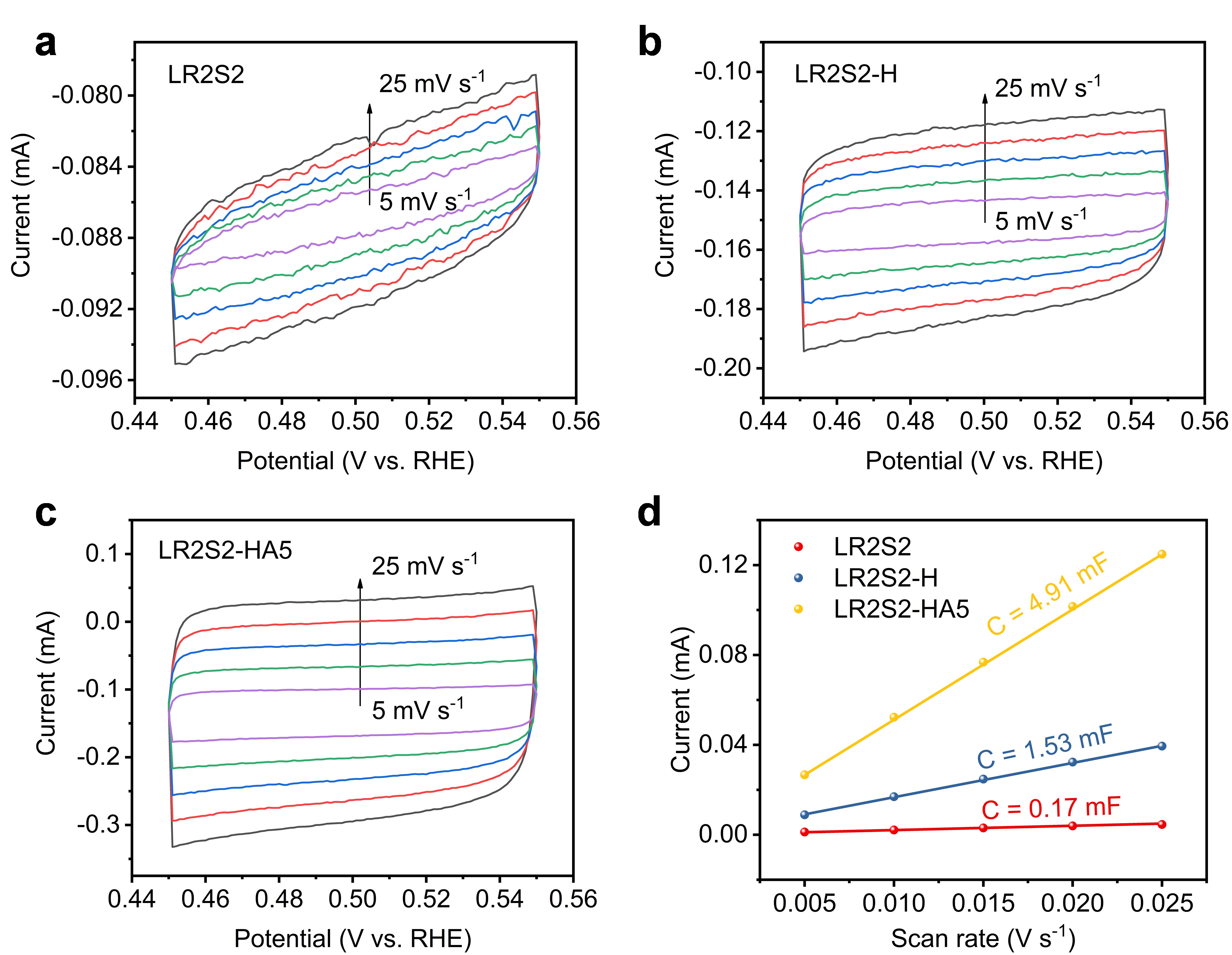


**Figure S6.** Double-layer capacitance (*C_dl_*) analysis. a) CV curves of LR2S2. b) CV curves of LR2S2-H. c) CV curves of LR2S2-HA5. d) *C_dl_* plots for LR2S2, LR2S2-H, and LR2S2-HA5.


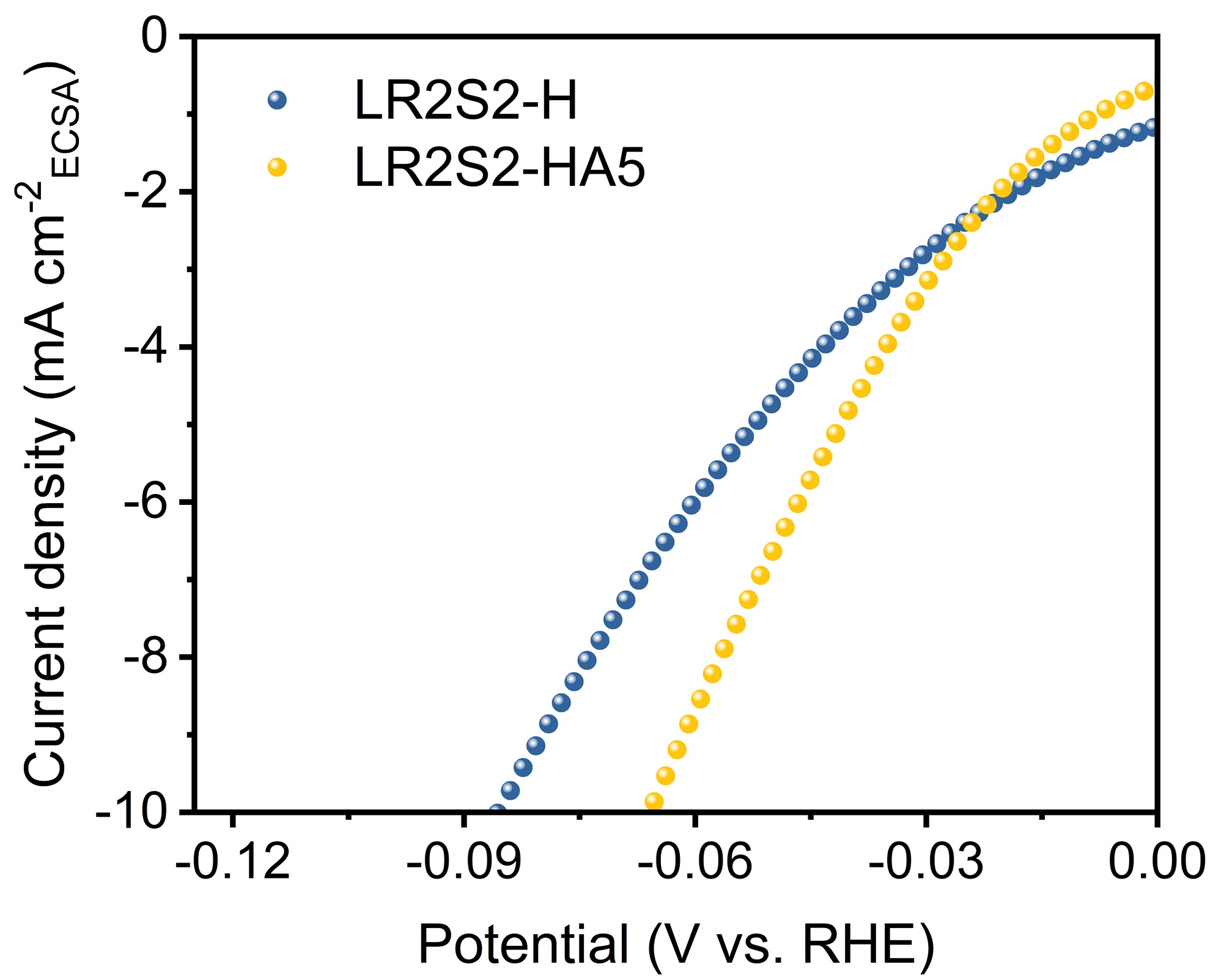


**Figure S7.** ECSA-normalized LSV curves of LR2S2-H and LR2S2-HA5.


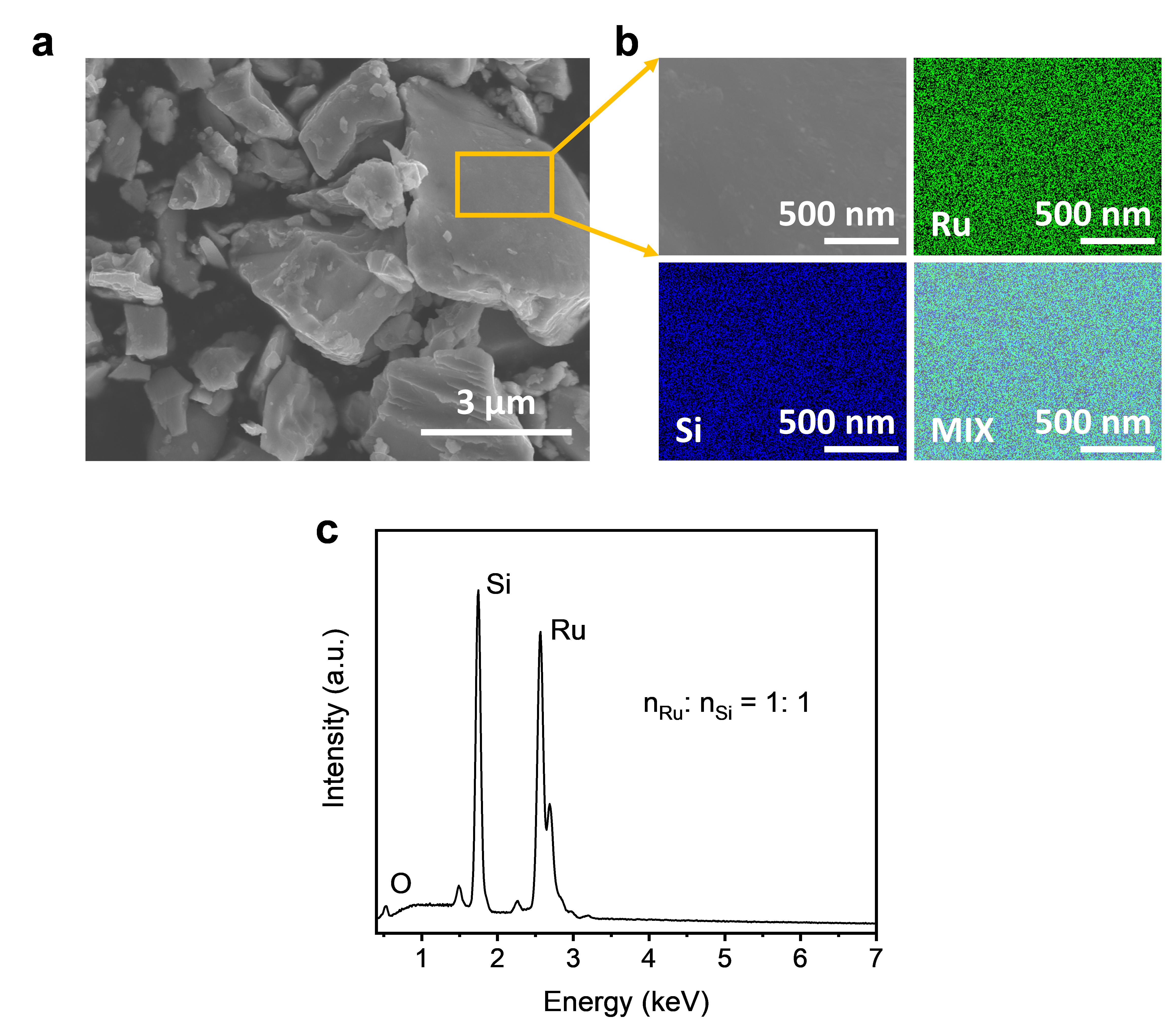


**Figure S8.** Morphology and elemental analysis of the RuSi intermetallic compound. a) SEM image. b) EDS Mapping. c) EDS spectrum. The results reveal a Ru:Si atomic ratio of approximately 1:1, with a minor oxygen presence attributed to surface-adsorbed oxygen species from air exposure.


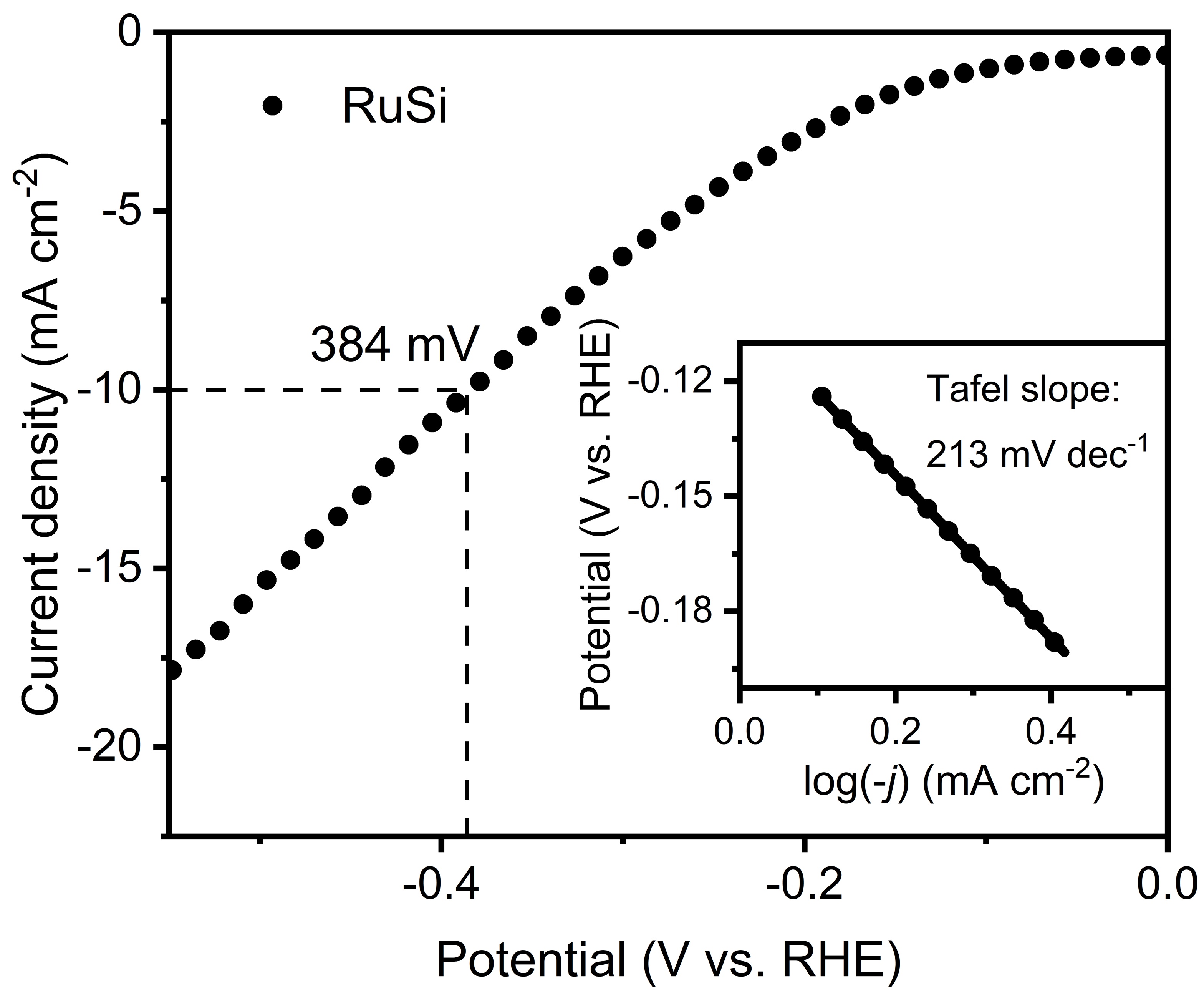


**Figure S9.** LSV curve of RuSi in 1 M KOH.


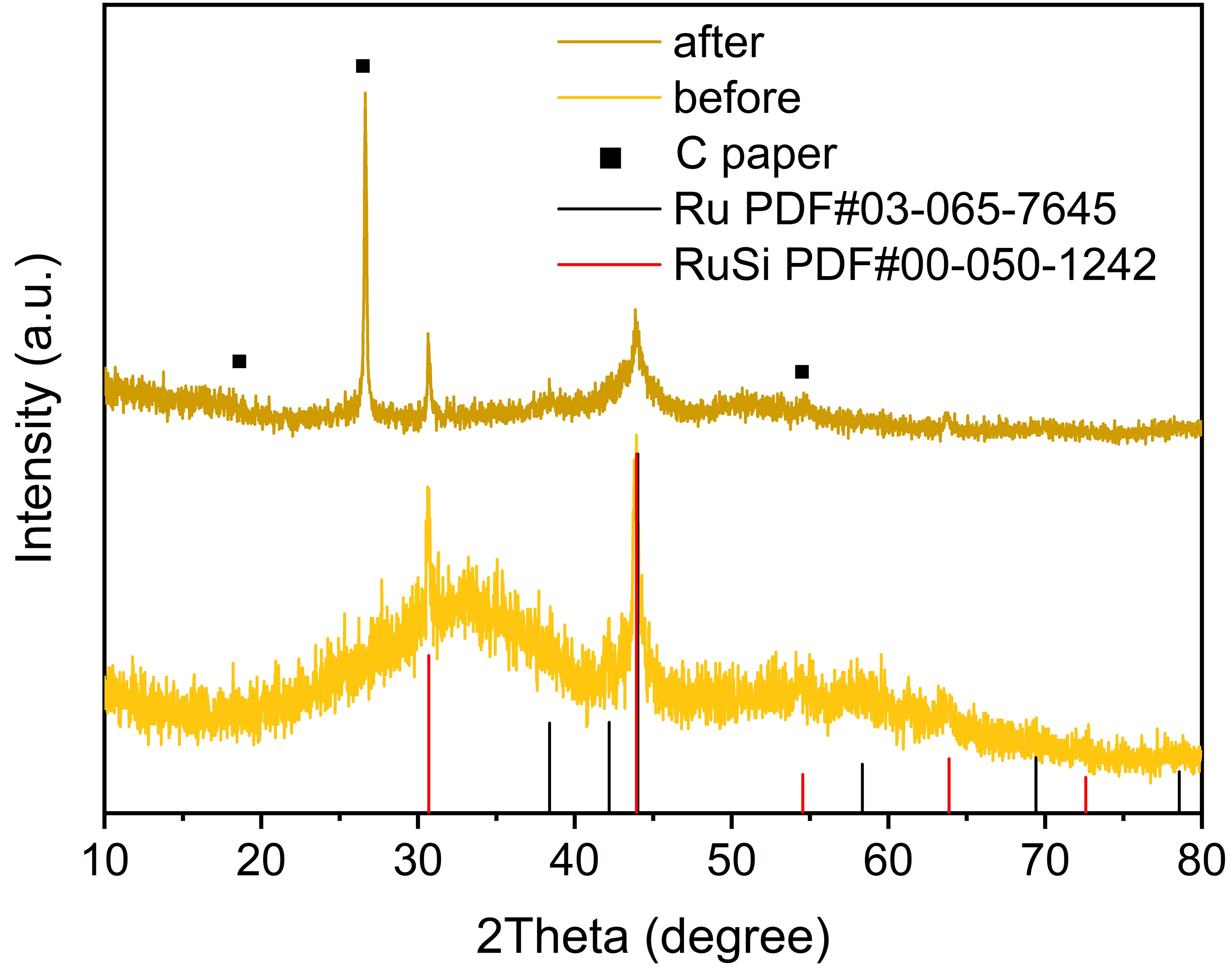


**Figure S10.** XRD patterns of the samples before and after catalytic reactions at 100 mA cm^-2^ for 10 hours.


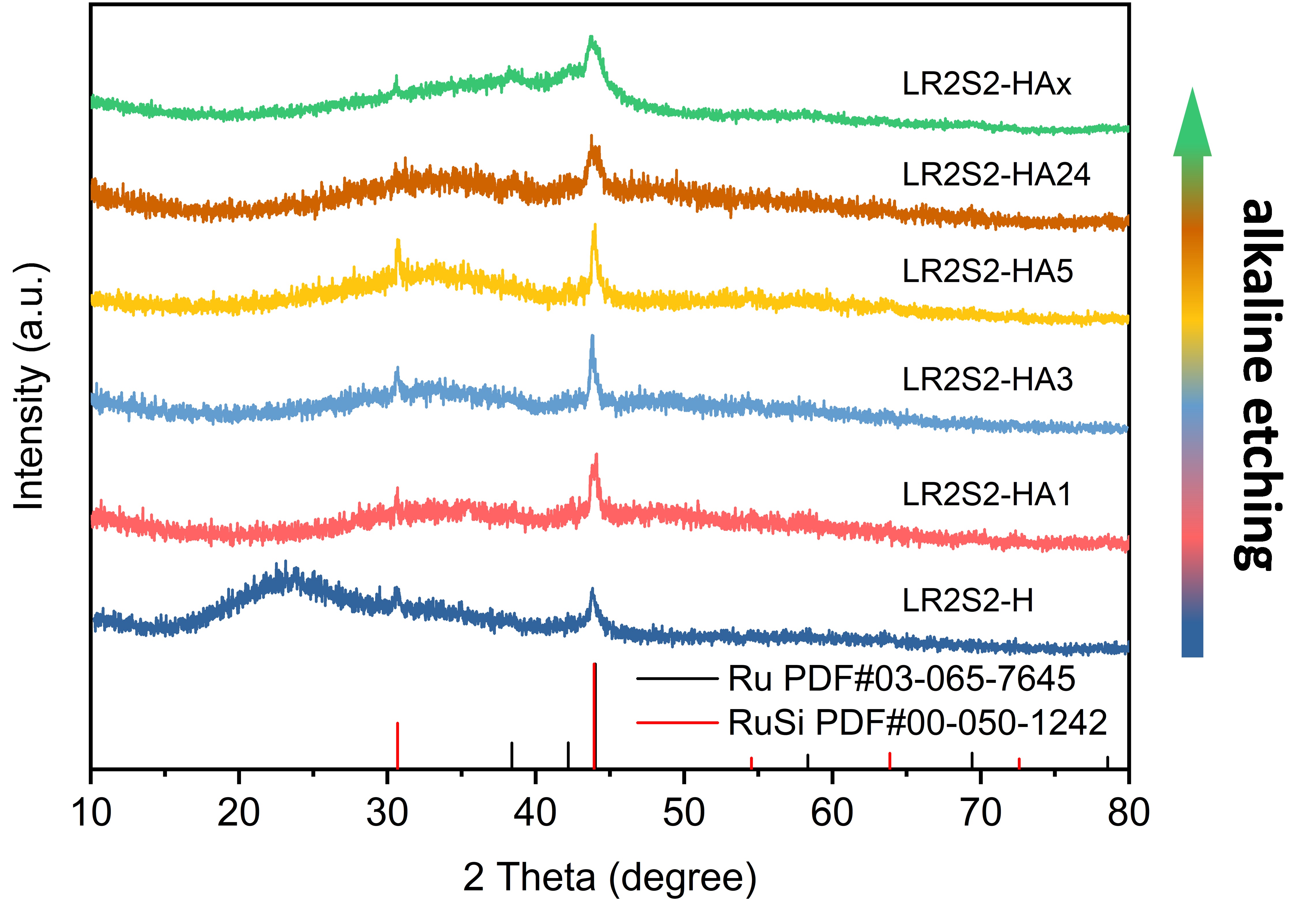


**Figure S11.** XRD patterns of samples with varying degrees of alkaline etching.


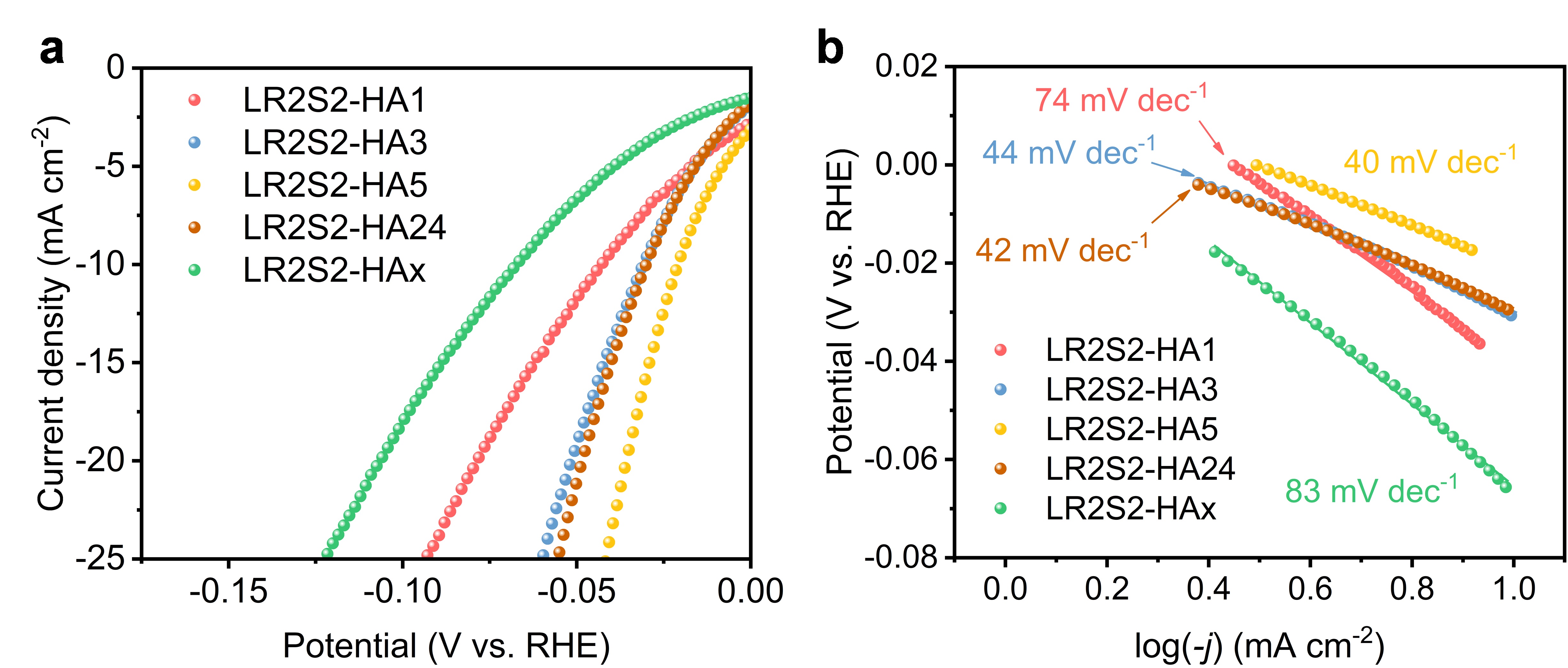


**Figure S12.** HER performance of samples with varying degrees of alkaline etching. a) LSV curves. b) Tafel slopes.


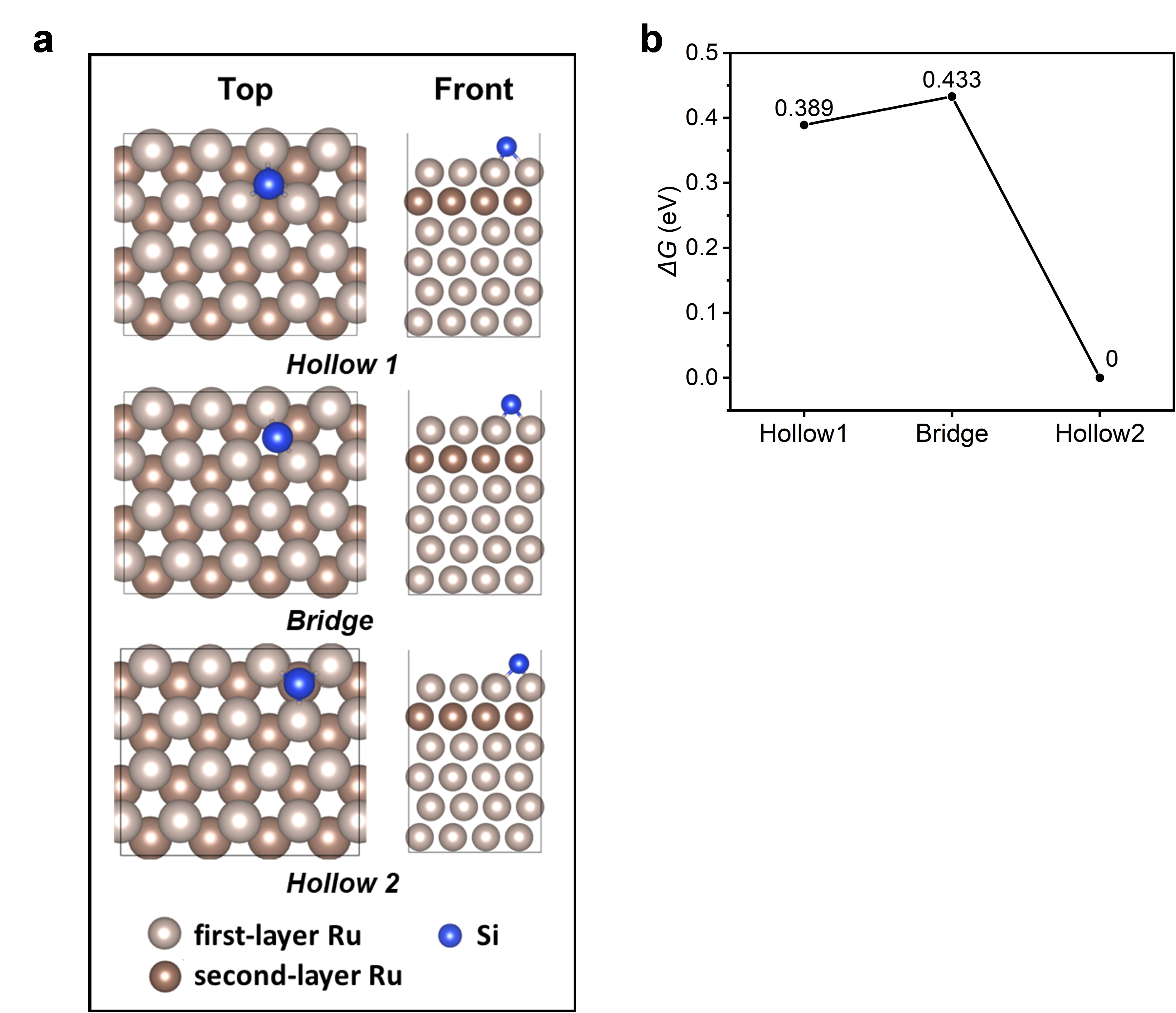


**Figure S13.** Adsorption of Si on the Ru (001) surface. a) Schematic illustration of Si adsorption on the Ru substrate. b) Si adsorption energy at different sites. These results indicate that the Hollow 2 site is the most stable site for Si adsorption. Therefore, this site was considered for Si adsorption in our rest calculations.

**
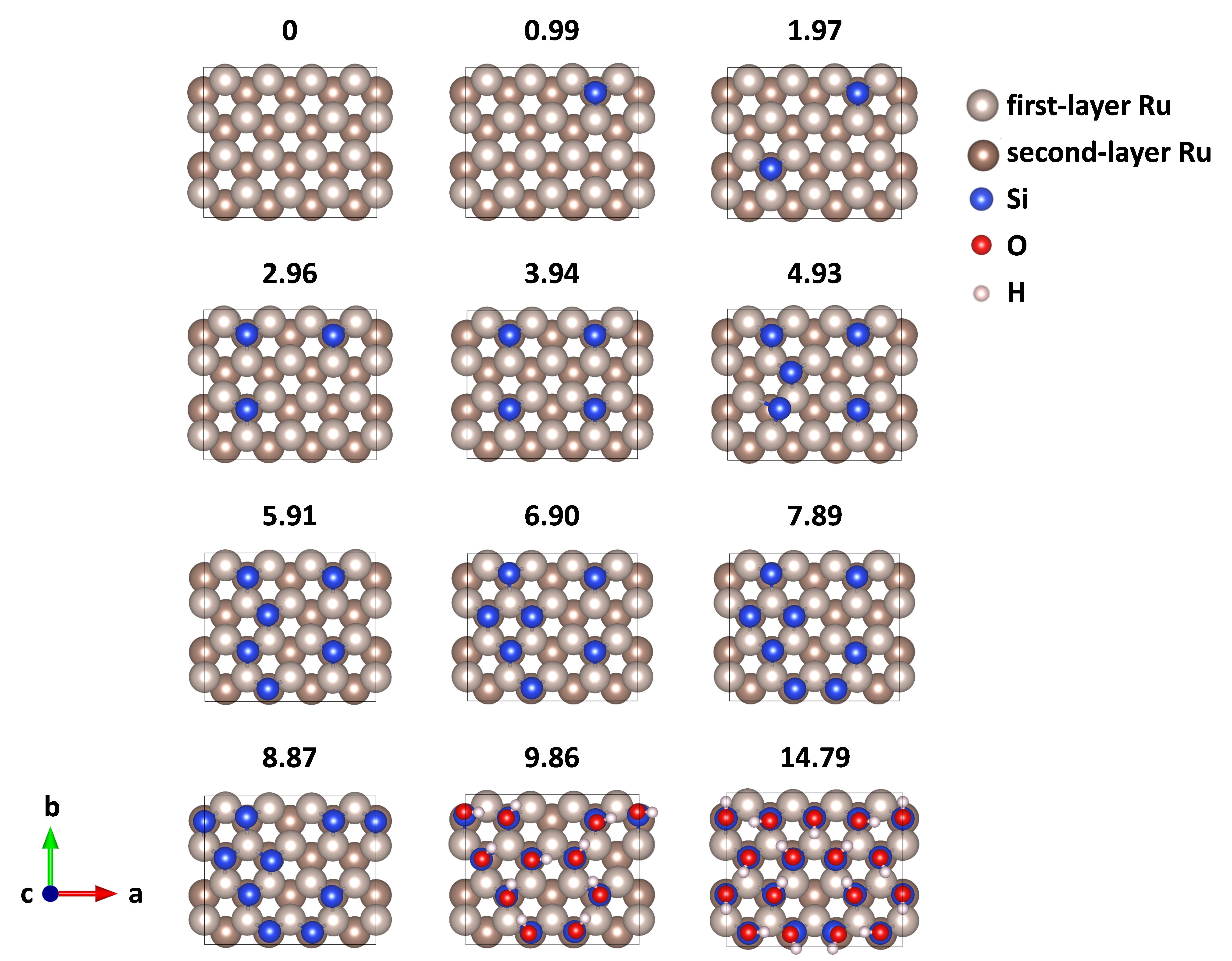
**

**Figure S14.** Top view of the optimized structures of the Si-decorated Ru (001) surface. The concentration (number nm^-2^) of adsorbed Si or Si-OH is indicated at the top of each structure.


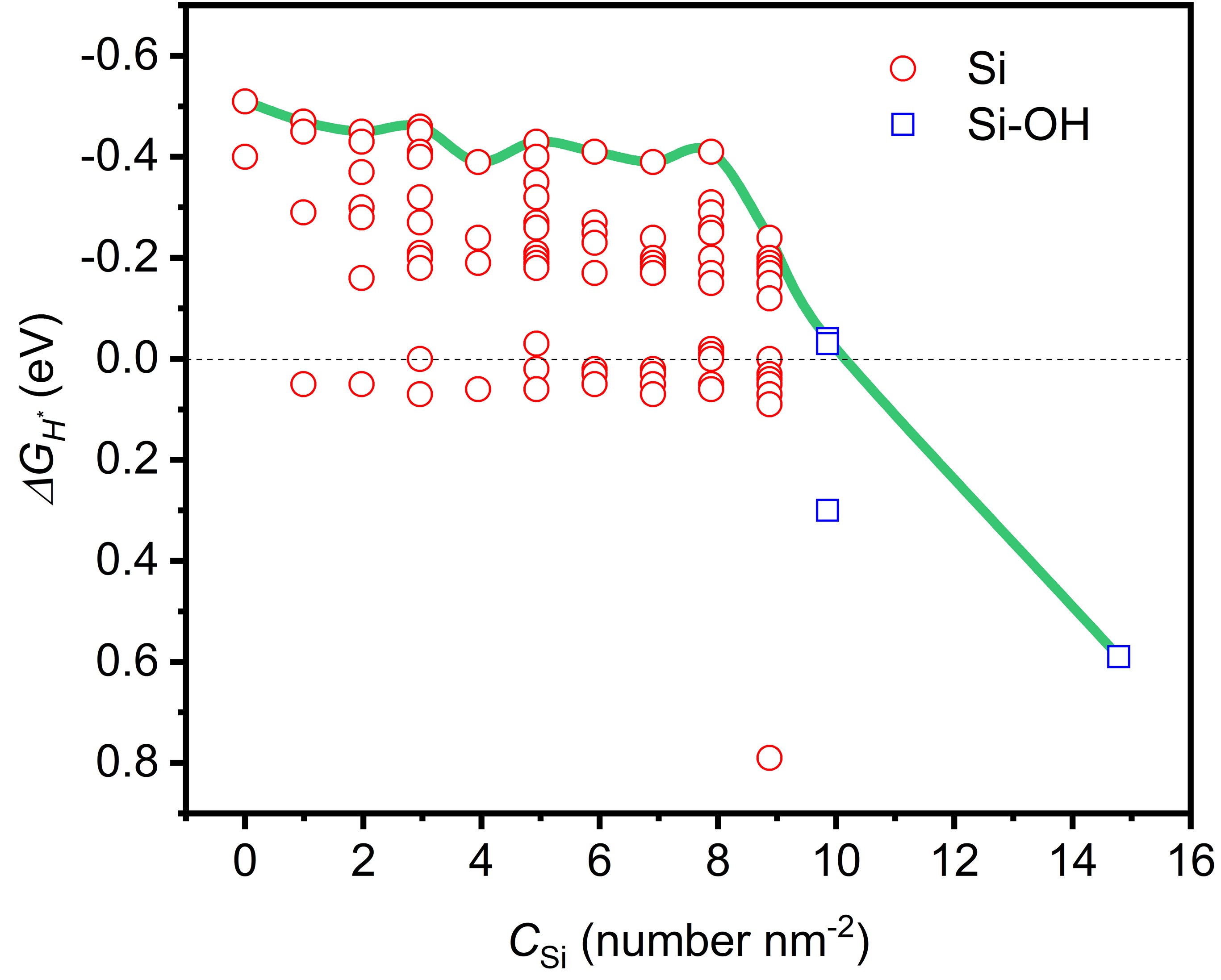


**Figure S15.** Dependence of H adsorption energy on Si concentration. The calculations were performed for multiple independent Ru sites, with the green line indicating the energetically most favorable H adsorption.


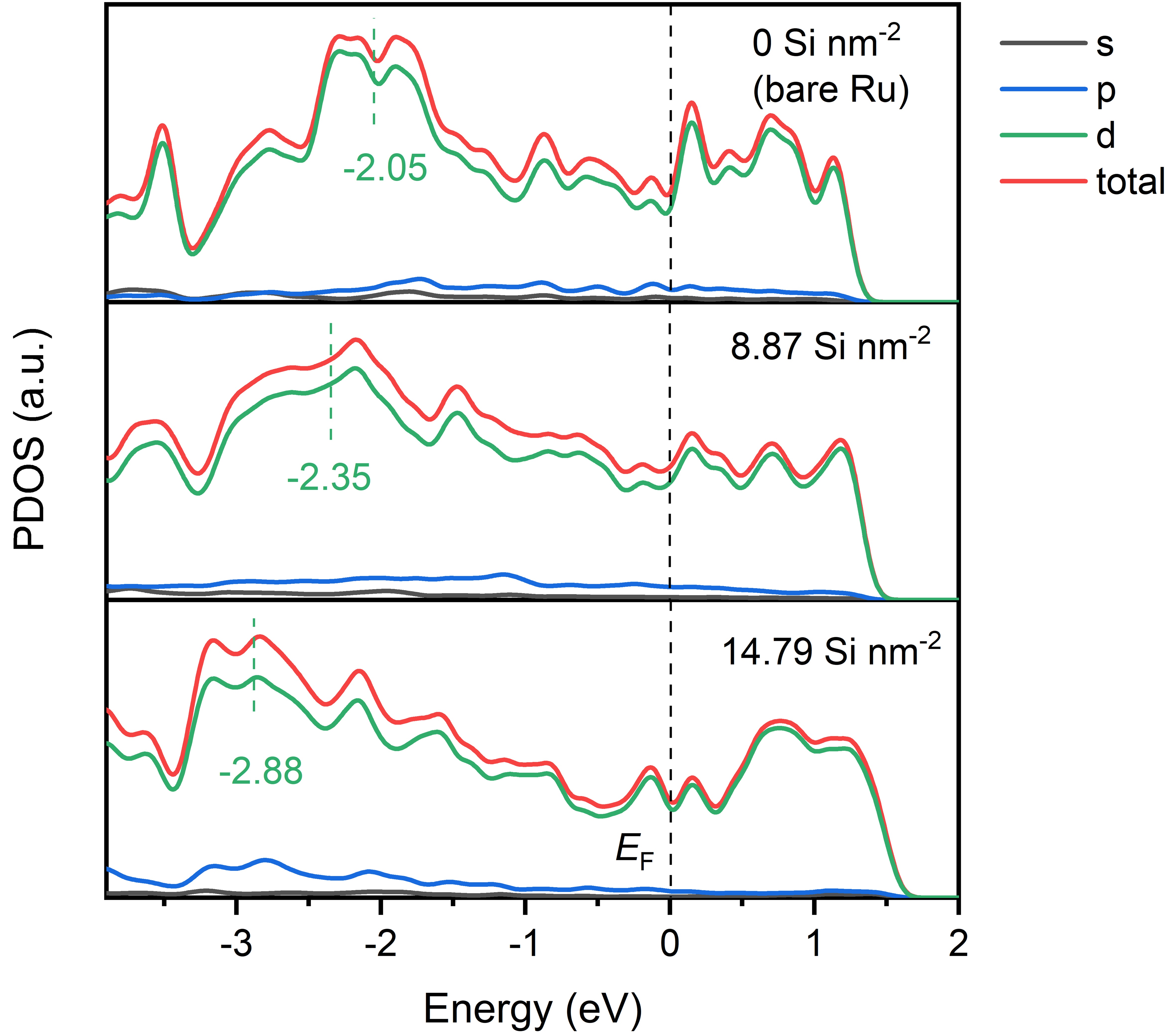


**Figure S16.** Projected density of states (PDOS) for surface Ru atoms. The Fermi level is set to zero and the green dashed line indicates the *d*-band center.


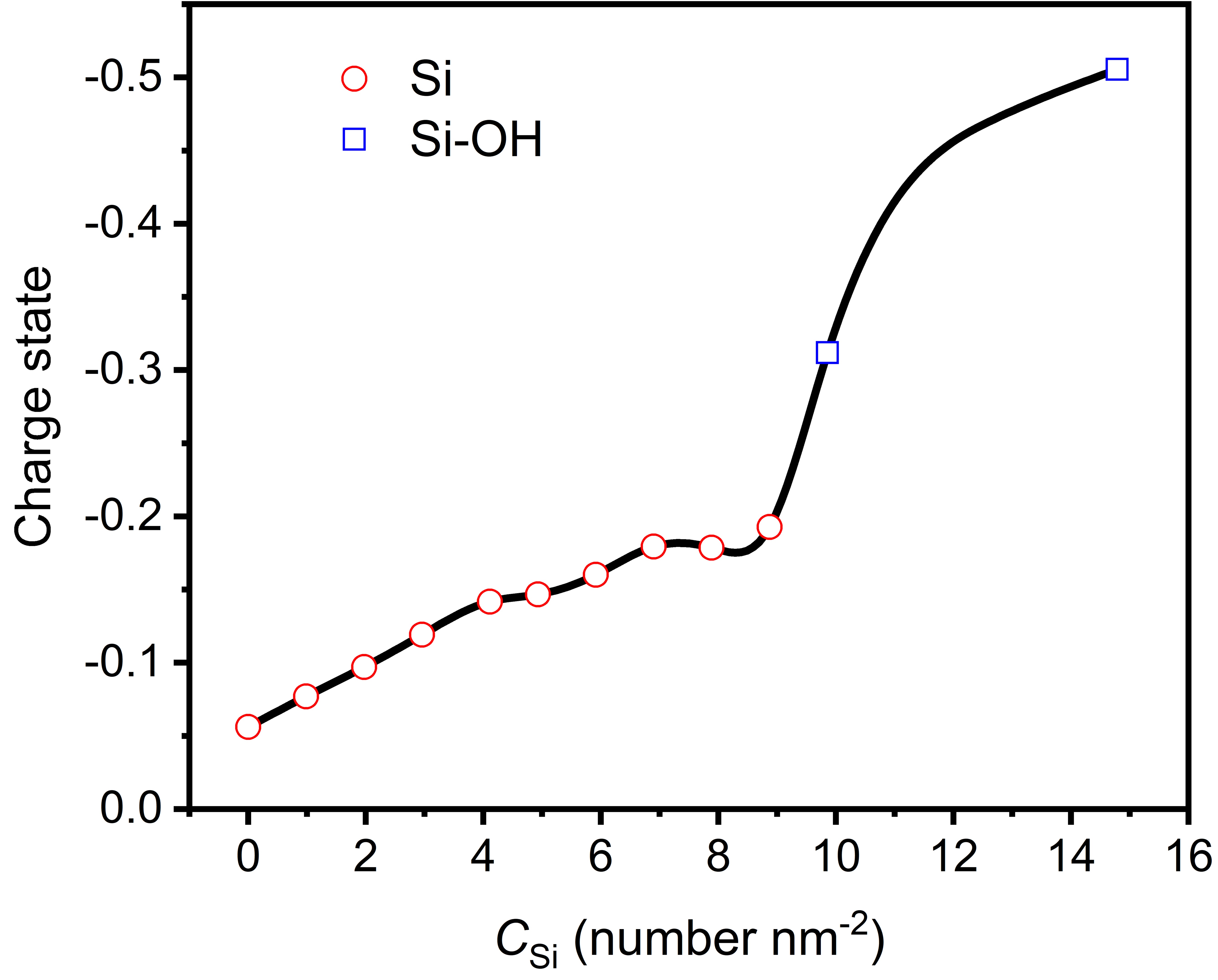


**Figure S17.** Average charge state of each surface Ru atom as a function of Si coverage.

**Table S1.** Comparison of HER performance of LR2S2-HA5 with reported electrocatalysts in 1 M KOH.

| **Electrocatalyst** | **Overpotential @10 mA cm^−2^**  **(mV)** | **Tafel slope**  **(mV dec^−1^)** | **References** |
| --- | --- | --- | --- |
| LR2S2-HA5 | 21 | 40 | This work |
| Pt/C  (Commercial catalyst) | 33 | 54 | This work |
| Ru/C  (Commercial catalyst) | 72 | 77 | This work |
| PtRu@C_2_N | 59 | 63 | *Chem. Eng. J.* 2022^[5]^ |
| Ru@NC | 26 | 36 | *Angew. Chem. Int. Ed.* 2018^[6]^ |
| Ni_5_P_4_-Ru | 54 | 52 | *Adv. Mater.* 2020^[7]^ |
| LaRuSi | 72 | 68 | *Angew. Chem. Int. Ed.* 2022^[8]^ |
| a-RuTe_2_ PNRs | 36 | 36 | *Nat. Commun.* 2019^[9]^ |
| LaRuSi_3_@Ru | 45 | 58 | *Adv. Funct. Mater.* 2024^[10]^ |
| CeRuSi-EK | 28 | 24 | *ACS Catal.* 2023^[11]^ |
| LaNi_0.5_Co_0.5_Ru | 43 | 70 | *Angew. Chem. Int. Ed.* 2024^[12]^ |
| RuS_2_ | 78 | 50 | *J. Mater. Chem. A* 2019^[13]^ |
| RuTe_2_ | 45 | 51 | *Small* 2021^[14]^ |
| RuAu-0.2 | 24 | 37 | *Adv. Energy Mater.* 2019^[15]^ |
| Br-Ru/RuP_2_ | 34 | 27 | *Adv. Mater.* 2024^[16]^ |
| RuS_x_/S-GO | 58 | 56 | *Small* 2019^[17]^ |
| Ru-FeP-CoP/NCP | 28 | 36 | *Carbon Energy* 2025^[18]^ |

**References**

[1] G. Kresse, J. Furthmüller, *Phys. Rev. B* **1996**, *54*, 11169.

[2] J. P. Perdew, K. Burke, M. Ernzerhof, *Phys. Rev. Lett.* **1996**, *77*, 3865.

[3] S. Grimme, S. Ehrlich, L. Goerigk, *J. Comput. Chem.* **2011**, *32*, 1456.

[4] P. E. Blöchl, *Phys. Rev. B* **1994**, *50*, 17953.

[5] C. Li, L. Zhang, Y. Zhang, Y. Zhou, J. Sun, X. Ouyang, X. Wang, J. Zhu, Y. Fu, *Chem. Eng. J.* **2022**, *428*, 131085.

[6] Z.-L. Wang, K. Sun, J. Henzie, X. Hao, C. Li, T. Takei, Y.-M. Kang, Y. Yamauchi, *Angew. Chem. Int. Ed.* **2018**, *57*, 5848.

[7] Q. He, D. Tian, H. Jiang, D. Cao, S. Wei, D. Liu, P. Song, Y. Lin, L. Song, *Adv. Mater.* **2020**, *32*, 1906972.

[8] S. Shen, Z. Hu, H. Zhang, K. Song, Z. Wang, Z. Lin, Q. Zhang, L. Gu, W. Zhong, *Angew. Chem. Int. Ed.* **2022**, *61*, e202206460.

[9] J. Wang, L. Han, B. Huang, Q. Shao, H. L. Xin, X. Huang, *Nat. Commun.* **2019**, *10*, 5692.

[10] H. Zhang, K. Song, Z. Lin, Z. Wang, L. Zhang, S. Shen, L. Gu, W. Zhong, *Adv. Funct. Mater.* **2024**, *34*, 2405897.

[11] W. Cai, C. Zhou, X. Hu, T. Jiao, Y. Liu, L. Li, J. Li, M. Kitano, H. Hosono, J. Wu, *ACS Catal.* **2023**, *13*, 4752.

[12] S. Shen, H. Zhang, K. Song, Z. Wang, T. Shang, A. Gao, Q. Zhang, L. Gu, W. Zhong, *Angew. Chem. Int. Ed.* **2024**, *63*, e202315340.

[13] Y. Zhu, H. A. Tahini, Y. Wang, Q. Lin, Y. Liang, C. M. Doherty, Y. Liu, X. Li, J. Lu, S. C. Smith, C. Selomulya, X. Zhang, Z. Shao, H. Wang, *J. Mater. Chem. A* **2019**, *7*, 14222.

[14] Z. Zhang, C. Jiang, P. Li, K. Yao, Z. Zhao, J. Fan, H. Li, H. Wang, *Small* **2021**, *17*, 2007333.

[15] C.-H. Chen, D. Wu, Z. Li, R. Zhang, C.-G. Kuai, X.-R. Zhao, C.-K. Dong, S.-Z. Qiao, H. Liu, X.-W. Du, *Adv. Energy Mater.* **2019**, *9*, 1803913.

[16] Z. Wu, Q. Li, G. Xu, W. Jin, W. Xiao, Z. Li, T. Ma, S. Feng, L. Wang, *Adv. Mater.* **2024**, *36*, 2311018.

[17] P. Li, X. Duan, S. Wang, L. Zheng, Y. Li, H. Duan, Y. Kuang, X. Sun, *Small* **2019**, *15*, 1904043.

[18] Y. Long, L. Yang, M. Xi, Y. Zhao, H. Zhang, T. Liu, A. Chen, X. An, G. Hu, Z. Ni, *Carbon Energy* **2025**, *7*, e690.
